# *Cistus ladanifer* L.: Essential Oils, Volatiles, By-Products, and Their Biological Properties

**DOI:** 10.3390/molecules30224425

**Published:** 2025-11-16

**Authors:** Custódia Gago, Boulanouar Bakchiche, Tahar Djekhioua, Maria da Graça Miguel

**Affiliations:** 1 MED—Mediterranean Institute for Agriculture, Environment and Development & CHANGE-Global Change and Sustainability Institute, Faculdade de Ciências e Tecnologia, Universidade do Algarve, Edf. 8, Campus de Gambelas, 8005-139 Faro, Portugal; 2 Laboratory of Biological and Agronomic Sciences (LBAS), University of Laghouat, Laghouat 03000, Algeria; b.bakchiche@lagh-univ.dz (B.B.); aminebiochimiste25@gmail.com (T.D.); 3 Research Unit on Medicinal Plants (URPM), Laghouat 03000, Algeria

**Keywords:** rockrose, labdanum, essential oils, antimicrobial, antioxidant, herbicidal properties, cytotoxicity, bioactive by-products

## Abstract

*Cistus ladanifer* L., commonly known as gum rockrose, is a Mediterranean shrub of growing interest due to its valuable essential oils (EOs) and labdanum resin. This review synthesizes current knowledge on the chemical composition and biological activities of EOs and hydrolates from *C. ladanifer* across Mediterranean regions, with particular emphasis on Spain, Portugal, Morocco, and France. α-Pinene, viridiflorol, and camphene were found to be the major constituents in the EOs with antioxidant and antimicrobial properties. Additionally, the identified biological properties have prompted studies exploring innovative strategies such as nanoparticle encapsulation, the development of bioactive films, and the incorporation of EOs into food and pharmaceutical packaging. By-products from EO distillation, including lignocellulosic residues, the extraction of phenolic-rich compounds, and hydrolates, have shown potential for value-added applications. Altogether, *C. ladanifer* represents a versatile species with possible applications in cosmetics, pharmaceutical development, and the food industry.

## 1. Introduction

*C. ladanifer* is the most economically important species in the fragrance and cosmetics sectors among the Cistaceae family. This family comprises eight genera, with *Hudsonia*, *Lechea*, and *Crocanthemum* found in temperate regions of the Americas, while *Cistus*, *Fumana*, *Helianthemum*, *Halimium*, and *Tuberaria* are native to the Mediterranean region [1]. The family includes approximately 220 species, primarily heliophyte shrubs, subshrubs, and herbs that grow in open habitats with nutrient-poor soils [2]. The highest diversity of genera and species occurs within the Mediterranean floristic region. The genus *Cistus* is further divided into three subgenera: *Cistus*, *Halimioides*, and *Leucocistus* [1].

Within the subgenus *Leucocistus,* several species are recognized, including *C. ladanifer* and *C. laurifolius* (section Ladanium); *C. salviifolius*, *C. sintenisii*, *C. monspeliensis*, *C. populifolius,* and *C. psilosepalus* in the (section Ledonia); and *C. pouzolzii* (section Stephanocarpoidea) [3,4,5,6,7].

**Figure 1:**
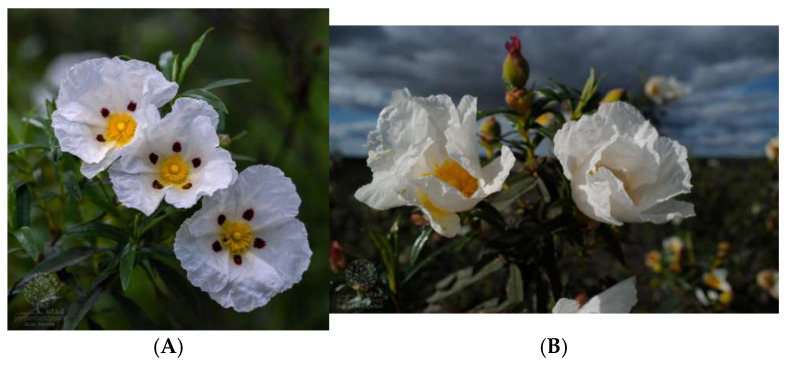
*Cistus ladanifer* subsp. *ladanifer* var. *maculatus* (**A**) and *Cistus ladanifer* subsp. *ladanifer* var. *albiflorus* (**B**).

Three subspecies are reported for *C. ladanifer*: subsp. *ladanifer* (Figure 1A,B), which is mainly distributed in the Iberian Peninsula, northern Africa, southern France, and isolated patches in the Canary Islands (being considered an introduced species in the two latter places); subspecies *sulcatus*, which was first identified as *Cistus palhinhae* and is endemic to southwestern Portugal (Algarve region); and subspecies *africanus,* which is found in southern Spain (Cádiz, Málaga) but is more frequently found in northern Africa [3].

*Cistus ladanifer* is a perennial and woody shrub that can form dense populations mostly integrating poor siliceous and acidic soils that are formed of sandstone, granite, or shale [8]. There are two varieties of *Cistus ladanifer* subsp. *ladanifer*: var. *maculatus*, which has a red to maroon blotch at the base of each petal (Figure 1A), and var. *albiflorus*, which has five petals that may be completely white (Figure 1B). These two varieties may coexist in populations that are mixed or can occur separated in monomorphic populations [3].

A: Author: Juan Sevilla of Jardim Botânico UTAD, Flora Digital de Portugal.

<p>Imagem da espécie <i>Cistus ladanifer subesp. ladanifer</i> por Juan Sevilla do <a href="https://jb.utad.pt" target="_blank">Jardim Botânico UTAD, Flora Digital de Portugal</a>.</p>

B: Author: Isabel Queiroz Marcellot of Jardim Botânico UTAD, Flora Digital de Portugal.

<p>Imagem da espécie <i>Cistus ladanifer subesp. ladanifer</i> por Isabel Queiroz Marcellot do <a href="https://jb.utad.pt" target="_blank">Jardim Botânico UTAD, Flora Digital de Portugal</a>.</p>

Among the Cistaceae family, *C. ladanifer* is the most economically significant species in cosmetics and fragrance industries. It is used to produce a range of aromatic products such as *Cistus* oil (concrete and absolute), raw labdanum gum, labdanum gum (concrete and absolute), and labdanum oil. Raw gum labdanum is a resin extracted from the photosynthetic leaves and stems of *C. ladanifer*, with alkaline water to remove all the surface waxes, followed by acid neutralization (method used in Andaluzia, Spain). In Zamora, León y Salamanca (Spain), the raw gum is obtained using boiling water, with a lower yield than the procedure of Andaluzia. The resin obtained through these methods comprises terpenoids, mainly labdane-type diterpenes, and methylated flavonoids [9,10,11,12]. Concrete can be extracted using organic solvents from the crude resin or from the leaves and stems of *C. ladanifer*. The labdanum gum can be extracted with ethanol to yield the absolute. Diterpenoid and flavonoid fractions can then be extracted from labdanum absolute [9]. Essential oils (EOs) that are extracted by steam distillation or hydro-distillation can, likewise, be extracted from the labdanum or the aerial parts of *C. ladanifer* [9,10].

The trichomes found in the adaxial and abaxial parts of the leaf, primarily in young, developing leaves, secrete the labdanum of *C. ladanifer*. The fact that trichome density differs amongst *C. ladanifer* populations indicates that biotic and environmental factors influence exudate production. Summertime is when exudate production is at its greatest since it is triggered by ultraviolet (UV) radiation and water stress [13]. This is also strengthened by its much greater production in the field in comparison with greenhouse plants [14]. The number of flavonoids present in the exudate varies noticeably with the season, with the summer months having the highest concentration and the winter months having the lowest. The seasons also affect the amount of diterpenes but in a different way, that is, the highest production is in the winter and the lowest in the spring and summer [15].

The reduced plant diversity and richness in the presence of *C. ladanifer* is believed to be due to the allelochemicals aglycone flavonoids and diterpenes, released by its leaves and stems and present in the exudates. The allelopathic potential of *C. ladanifer* is due to the joint action of several secondary metabolites that, in turn, is dependent on the environmental factors (season, temperature, and photoperiod) [13,16].

The labdanum, essential oil (EO), and other extracts of *C. ladanifer* are used as fixatives in fragrance and cosmetics. Its traditional use as a medicinal plant is mostly associated with its ability to heal wounds. The EO is used to treat wounds, skin ulcers, hemorrhages, secondary infections, and other skin conditions like psoriasis and eczema. In addition to its use on the skin, the EOs and/or extracts have been shown to have anti-ulcerogenic, anti-acid, anti-diarrheal, antispasmodic, diuretic, analgesic, hypoglycemic, antihypertensive, and vasodilator properties in various target organs [17]. *C. ladanifer* branches have been employed to draw and catch houseflies (*Musca domestica* L., Diptera: Muscidae) due to their strong scent. Because its leaves are sticky, bunches of this species are hung up and serve as fly-capture tape [18]. The repellent activity of *C. ladanifer* EO using the mosquito *Aedes aegypti* was revealed to be poor when compared with those of *Lavandula stoechas*, *Helichrysum italicum*, and *Laurus nobilis* [19].

This review aims to synthesize the current knowledge on the chemical composition and biological properties of the EOs and volatile compounds derived from *Cistus ladanifer*.

## 2. Methods

**Figure 2:**
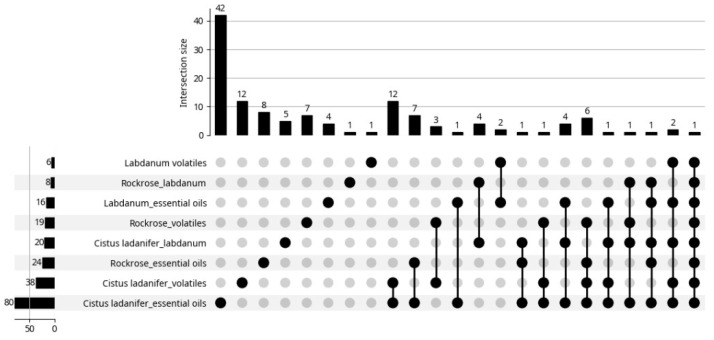
Distribution of the articles by sections. Google Gemini. (2025). Generation of article distribution chart by sections [Computer software]. Available from https://gemini.google.com**.** Accessed 13 June 2025.

**Figure 3:**
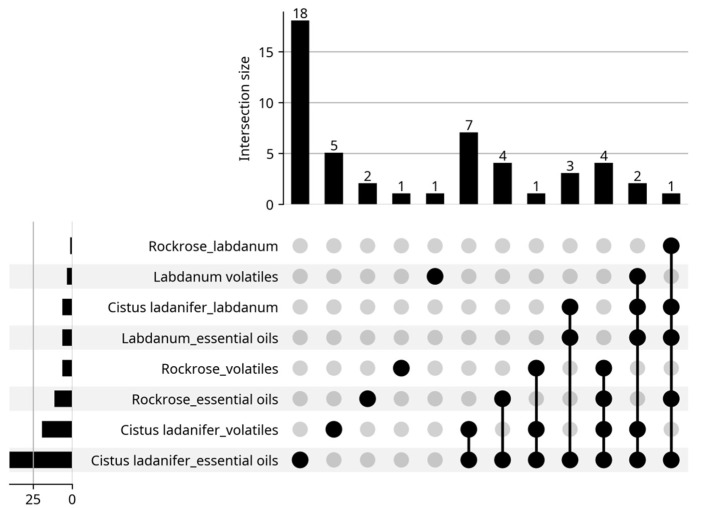
Distribution of articles about essential oils by sections. Google Gemini. (2025). AI-generated figure for article distribution analysis [Computer software]. Available from https://gemini.google.com.

The present work provides a brief review of the volatiles extracted from *Cistus ladanifer* as well as their properties and applications. The information in this review was taken from articles found on the Web of Science (WOS) platform, using the keywords “*Cistus ladanifer* essential oils”, “*Cistus ladanifer* volatiles”, “Rockrose essential oils”, “Rockrose volatiles”, “Labdanum essential oils”, “Labdanum volatiles”, up to 31 May 2025. A total of 211 results were obtained, distributed by the eight keywords (sections) as can be seen in the bottom left corner of Figure 2 (sum of all data). However, some articles appeared in several sections as depicted in the same Figure. In fact, instead of 211 articles, only 127 articles were really found (sum of all data over bars). For example, there is an article that appeared in all sections; it was a review article. After reading the articles, only 49 (Figure 3) addressed essential oils (46) or emission of volatiles (3 articles). Six of the forty-nine articles lacked the chemical composition of the essential oils. The biological properties of the rockrose EOs were the primary focus of these articles. The articles may appear in multiple sections, as expected, and the review is performed with the information provided by these articles (Figure 3).

## 3. Chemical Composition of Essential Oils

Since ancient times, plants that produce volatile compounds (aromatic plants) have been utilized as spices, medical treatments, and in religious rituals due to their therapeutic qualities and appealing scents [20].

Essential oils (EOs) are complex mixtures of volatile compounds made by living organisms that are extracted solely by physical methods (pressing and distillation) from entire plants or plant parts that are recognized to have a specific taxonomic origin [21]. Other methods of extracting volatile fractions from aromatic plants do not fit the official definition of “essential oils” [22].

Essential oils (EOs) are composed of a wide variety of volatile compounds, including terpenes with regular carbon skeletons such as monoterpenes (C_10_), sesquiterpenes (C_15_), and diterpenes (C_20_) [23,24]. Terpenoids with irregular carbon skeletons, such as homoterpenes and norisoprenoids, also contribute to EO composition [23]. Phenolics derived from shikimic pathways can also be constituents of EOs such as methyl salicylate, which is the major component of wintergreen, or anethole in anise and fennel [23].

**Figure 4:**
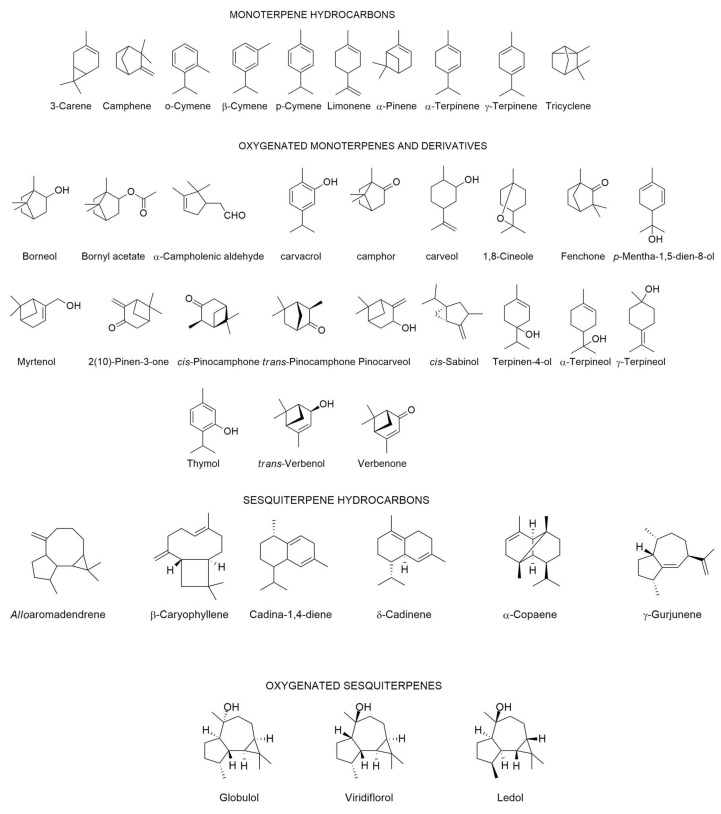

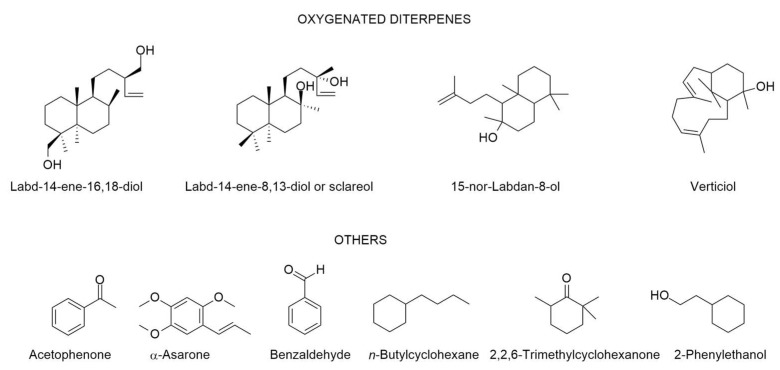
Structures of the main compounds (>4.5%) found in the *C. ladanifer* EOs. Sections may be divided by subheadings.

The chemical composition of *C. ladanifer* EO collected from different places in the Mediterranean area including Portugal is depicted in Table 1, since 1997. In the same Table, there is also the chemical composition in volatiles of mixtures obtained by methods other than hydro-distillation. It also described the composition of the volatile fraction of resinoids, concretes, and absolutes. In those cases where the authors had determined the biological properties (e.g., antimicrobial, antioxidant activities, among other ones), the results were also reported in the same Table. The structures of the main compounds (>4.5%) present in the *C. ladanifer* EOs are depicted in Figure 4.

### 3.1. Chemical Composition of Cistus ladanifer EOs from the Iberian Peninsula

Based on research published in the Web of Science (WOS) up to March 2025, Spain, Portugal, and Morocco have contributed the largest number of studies on the chemical composition of EOs or volatiles from *C. ladanifer*, with 13, 10, and 8 publications, respectively (Table 1).

In Spain, ledol [52], such as *trans*-pinocarveol [46], predominated in one sample, viridiflorol in three [53,55,56], and α-pinene in seven cases [45,47,48,49,50,51,54] (Table 1). Viridiflorol was found in six other samples even though it was not the main monoterpenoid. α-Pinene and viridiflorol appear to be typical of *C. ladanifer* volatiles or EOs from Spain (Table 1). Already in 1969, Garcia-Martin and Garcia-Vallejo [59] studied the *C. ladanifer* var. *maculatus* EO from Spain for the first time and reported that α-pinene was dominant (44%), followed by camphene, *p*-cymene, 1,8-cineole, borneol and linalyl, and bornyl acetate but at much lower percentages.

The chemical composition of the neutral volatile fraction of a commercial EO of *C. ladanifer* produced in Spain was evaluated by Simon-Fuentes et al. [60]. The fractionation of the EO was carried out by aqueous NaHCO_3_ and NaOH extraction and column chromatography that produced four fractions: carboxylic acids, phenols, mono-and sesquiterpene hydrocarbons, and oxygenated compounds. In the neutral volatile fraction, Simon-Fuentes et al. [60] found three main compounds, with concentrations of which percentages were >5%: α-pinene (35), camphene (10) and 2,2,6-trimethylcyclohexanone (6) [60].

The enantiomeric distribution of chiral compounds was determined for six pairs of the *C. ladanifer* EO from Spain by Costa et al. [45]. The enantiomeric distribution for the main compound, α-pinene, was 6.8 (−):93.2(+).

In six samples of *C. ladanifer* EOs from Portugal, α-pinene was the main monoterpene. One sample had 2,2,6-trimethylcyclohexanone, another viridiflorol [11,17,37,38,39,40,41,43,44], and one had acetophenone, which was obtained via sequential extraction using water and dichloromethane instead of steam distillation, possibly affecting the chemical composition [36] (Table 1).

In both Portugal and Spain, α-pinene is the main constituent of *C. ladanifer* EO, followed by viridiflorol in Spain and camphene in Portugal.

The variability in the chemical composition of the EOs can be attributed to several factors, including geographic location, seasonal variation, and plant age. For example, for plants collected in Andévalo (Huelva, Spain), α-pinene (42.50%) was the main constituent of the EO followed by viridiflorol (13.36%), whereas for the EO obtained from plants of Cerezal (Zamora, Spain), the concentrations of these two monoterpenes were much closer (19.27 and 24.13%, respectively) [49].

**Figure 5:**
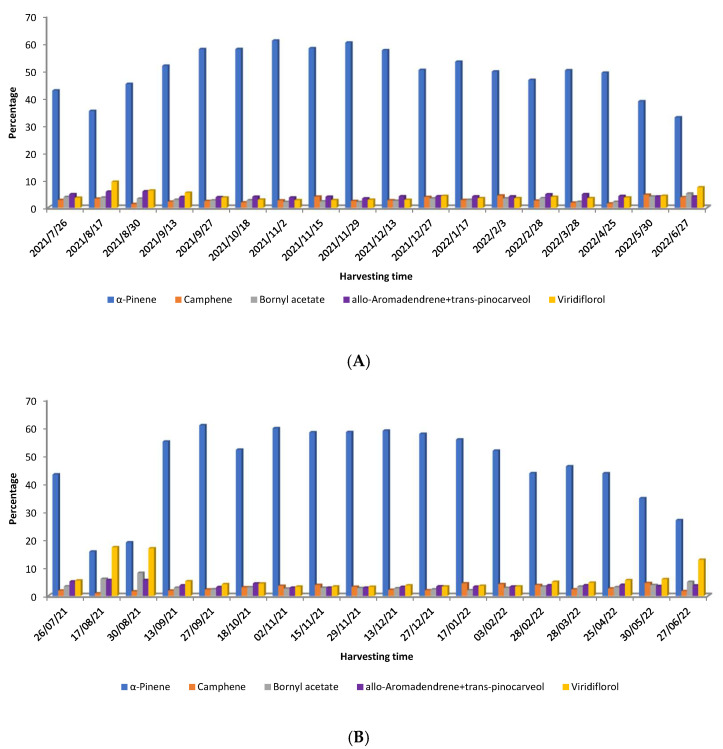
Distribution of the main compounds (>4.5%) of *C. ladanifer* EO obtained from 7-year-old plants from Bustares (**A**) and from 12-year-old plants from Hiendelaencina (**B**) [54].

Chaloupková et al. [54] studied the variation in yield and chemical composition of *C. ladanifer* EOs obtained from two locations in central Spain over one year and obtained from steam distillation. The age of plants was also different in those two zones. The oil yield ranged from 0.03 to 0.19% (weight/dry weight), being higher during the autumn and early winter months. Regarding the chemical composition, the authors [54] observed that a variation also occurred over time in both samples. The pattern of the main compounds (>4.5%) is depicted in Figure 5A (7-year-old plants from Bustares) and 5B (12-year-old plants from Hiendelaencina).

α-Pinene was predominant in both plant samples, but its biosynthesis varied seasonally (Figure 5). In August and May–June (Figure 5A,B), lower α-pinene and higher viridiflorol levels were observed, especially in older plants. These monoterpenes showed an inverse relationship. In 12-year-old plants, α-pinene and viridiflorol levels were nearly equal in August (Figure 5A,B). Despite differences in age and harvesting location (Hiendelaencina for older plants, Bustares for younger), the accumulation of α-pinene and viridiflorol followed a similar pattern across seasons (Figure 5A,B) [54].

**Figure 6:**
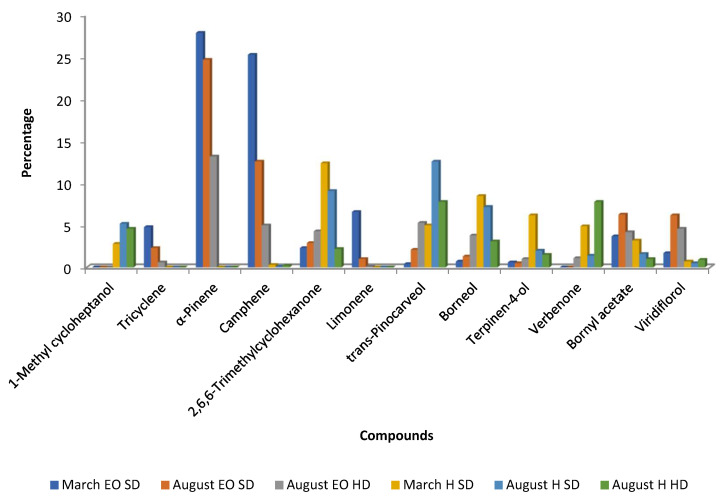
Main compounds (>4.5%) of *C. ladanifer* essential oil (EO) and hydrolates (H) obtained by steam distillation (SD) or hydro-distillation (HD) from wastes collected in Beira Baixa (Portugal) in March and August [42].

Essential oils obtained from *C. ladanifer* waste materials in Beira Baixa (Portugal) during March and August revealed notable seasonal differences in composition: α-pinene levels were higher in March, whereas viridiflorol was more abundant in August (Figure 6) [42]. This finding is somehow similar to that observed in samples from Spain, using the same extraction procedure (steam distillation for 1 h in samples from Spain and 1 h 30 min in Portugal). It seems that α-pinene is preferentially biosynthesized in March (winter/spring) and viridiflorol in August (summer). Figure 6 also depicts that monoterpene hydrocarbons (tricyclene, α-pinene, camphene, and limonene) decreased from March to August, in contrast to viridiflorol, bornyl acetate, *trans*-pinocarveol, and borneol, as well as the ketone 2,2,6-trimethylcyclohexanone (Figure 6). In the same work, these authors also compared the chemical composition of the EOs obtained by steam distillation and hydro-distillation, and the results are presented in Figure 6. The EOs obtained by hydro-distillation had less amounts of α-pinene, camphene, limonene, bornyl acetate, and viridiflorol, and higher concentrations of *trans*-pinocarveol, borneol, verbenone, terpinen-4-ol, and 2,2,6-trimethylcyclohexanone than those oils obtained by steam distillation (Figure 6). These differences can be the result of two factors: type of extraction and extraction time because in the hydro-distillation the time of extraction was 3 h in contrast to 1 h or 1 h 30 min in steam distillation [42,54].

**Figure 7:**
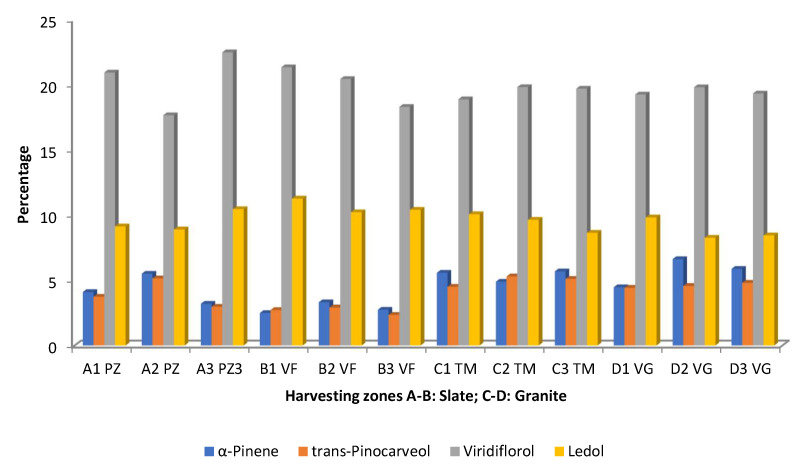
Main compounds (>4.5%) of *C. ladanifer* (EO) obtained from plant populations (A1,B1,C1,D1; A2,B2,C2,D2; and A3,B3,C3,D3 growing in PZ: Pozuelo de Zarzón (slate substrate); VF: Valverde del Fresno (slate substrate); TM: Torre de Don Miguel (granite substrate); VG: VillasBuenas de Gata (granite substrate) [53].

The effects of collection zone considering the edaphic substrate, and the intra-population variability were studied by Pérez-Izquierdo et al. [53]. The results are summarized in Figure 7, in which only the compounds present at >4.5% are represented. Viridiflorol and ledol were the predominant compounds. The variability in the amounts of viridiflorol is higher in the populations growing in slate substrates because it is possible to have samples with the highest percentage (A3 PZ) and the lowest percentage (A2 PZ) (Figure 7). In this case, there is also intra-population variability. The granite substrate does not exhibit this diversity as much (Figure 7). The populations B1–B3 had higher percentages of ledol in a consistent form, while the remaining samples showed variations between the populations and substrates. The EOs isolated from the populations A3 and B1–B3 had the lowest amounts α-pinene and *trans*-pinocarveol (Figure 7). All samples of the granite substrates along with the samples A1 and A2 had the highest percentages of those compounds. Once more, compared with plants growing in substrate slate, the EO samples taken from plants growing in granite substrate were more uniform (Figure 7).

**Figure 8:**
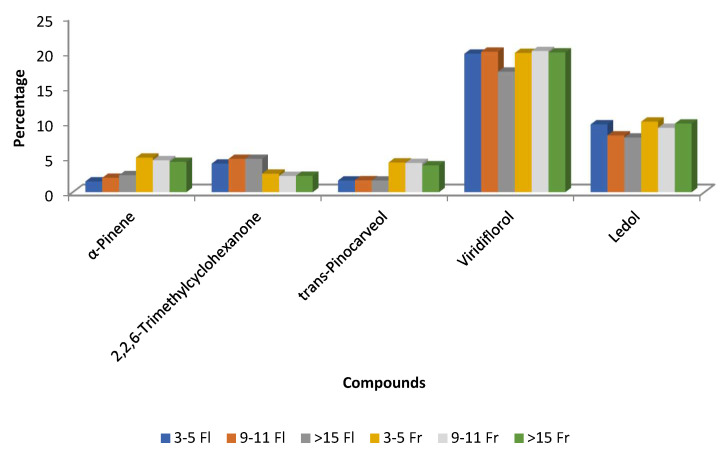
Main compounds (>4.5%) of *C. ladanifer* (EO) obtained from different aged plants at two phenological stages in Extremadura (Spain). 3–5 Fl: plants of 3–5 years old at flowering phase; 9–11 Fl: plants of 9–11 years old at flowering phase; >15 Fl: plants of >15 years old at flowering phase; 3–5 Fr: plants of 3–5 years old at fruit maturation phase; 9–11 Fr: plants of 9–11 years old at fruit maturation phase; > 15 Fr: plants of >15 years old at fruit maturation phase [55].

In two studies, Pérez-Izquierdo and co-authors examined the impact of the phenological stage on the chemical composition of *C. ladanifer* EOs from northwest Extremadura. In the first study, viridiflorol and ledol were more prevalent in August (20.38% and 8.92%, respectively) than in October (15.97% and 7.26%), whereas α-pinene was greater in October (12.91%) than in August (3.38%) [56]. In the second study, EOs from plants harvested in May (flowering phase) and August (fruit maturation) at several ages (3–5, 9–11, and >15 years) were assessed. Viridiflorol and ledol levels were lower in EOs from plants older than 15 years during flowering than in those during the fruit maturation stage. During the fruit maturity period, there was a decrease in 2,2,6-trimethylcyclohexanone and an increase in *trans*-pinocarveol and α-pinene (Figure 8) [55]. Regarding EO yields, *C. ladanifer* EO yield values ranged from 0.11 to 0.42 mL/100 g of dry plant material. The average EO yield of plants harvested at the flowering phenological stage was substantially lower (0.17%) than that of plants picked at fruit maturation (0.27%). Moreover, plants between the ages of three and five produced noticeably less than those between the ages of 9 and 11 at the flowering stage. However, no significant differences in EO yield were observed for the three age ranges studied at the fruit maturation stage [55].

Gomes et al. [37] reported that viridiflorol predominated in all EOs of *C. ladanifer* samples (wild plants collected in the center-interior of Portugal and cultivated plants in the north of Portugal that were propagated from a wild plant found in the dry plain region in the South of Portugal); nevertheless, 15-nor-labdan-8-ol was at higher concentrations in the EOs obtained from the cultivated plants (5.2%) than in the wild ones (1.7%). Small differences were also observed in the chemical composition of the EOs obtained from dried and fresh material (Table 1).

The effect of plant storage on the chemical composition of the essential oil of *C. ladanifer* was evaluated by Mediavilla et al. [50] in plants collected in Hiendelaencina—Guadalajara (Spain). The results showed that after 120 days of storage, a decrease in α-pinene (49.64–46.67%) and an increase in viridiflorol (10.03–12.50%) were observed. The boiling points of viridiflorol (293.0–294.0 °C) and α-pinene (155–156 °C) may help to explain these results. Despite the decrease in the yield of EO obtained from stored *C. ladanifer* plants over time, the authors [50] reported that this difference could not be considered significant at the 95% confidence level (Table 1). They considered a normal variability between bales, related to variations in the proportion of young twigs and wood, rather than because of the time elapsed after harvesting [50].

Using supercritical CO_2_, Rincón et al. [57] isolated volatiles from *C. ladanifer* leaves collected in July in Ciudad Real, Spain. They investigated how the chemical composition was affected by temperature, pressure, and extraction time. Regardless of extraction duration, camphor and α-pinene were the most abundant chemicals, followed by camphene, borneol, γ-terpineol, and thymol. The authors found that the yields dropped with temperature (40–60 °C), although there was no significant difference between 30 and 40 °C. Oil yields increased as pressure rose (8–10 MPa), with a significant increase between 8 and 9 MPa but less so between 9 and 10 MPa. The experimental data revealed a similar pattern at 30, 50, and 60 °C [57].

In order to distinguish between *C. ladanifer*, *Pinus pinaster* EOs, and *C. ladanifer* oil adulterated with *P. pinaster*, *Melaleuca alternifolia*, and red fruits, Viciano-Tudela et al. [61,62] tested MQ sensors with microcontrollers. They found that KNN (k-nearest neighbors) attained 100% accuracy with a precision and recall of 1 compared with support vector machine (SVM), naive Bayesian (NB), and neural network (NN) algorithms. Later on, Blasco et al. [63] reported 91.6% accuracy for terpenic hydrocarbons utilizing MQ sensors but limited accuracy for viridiflorol and α-pinene. This was the first time that metal oxide sensors were used to accurately determine the concentration of a chemical constituent in a complex EO matrix. The authors [61,62,63] working on this subject concluded that this technique provided an inexpensive means of evaluating EO quality and identifying adulteration.

*Cistus ladanifer* collected in Spain and its EO obtained by hydro-distillation was also chemically studied but using different approaches. The authors [64] used attenuated total reflectance Fourier-transform infrared (ATR-FTIR) vibrational spectroscopy, *differential scanning calorimetry* (DSC), and thermogravimetric (TG) analyses. The results showed that the specific location of the bands from unsaturated and α,β-unsaturated oxo groups had been related to the different contents of terpenoids of *C. ladanifer* EO (mono- and sesquiterpenoids). With regard to thermal behavior, *C. ladanifer* EO showed relatively high thermal stability, leading the authors [64] to conclude that EO constituents were conserved in the extraction process, in this case hydro-distillation.

### 3.2. Chemical Composition of Cistus ladanifer EOs from Morocco

The chemical profiles of *C. ladanifer* EOs from Morocco differed from those of the Iberian Peninsula. Three samples were dominated by viridiflorol [53,54,55], two by camphene [31,32], and one sample each by γ-terpinene [35], 1,8-cineole [30], and *trans*-pinocarveol [28] (Table 1).

The chemical composition of the varieties *maculatus* [28] and *albiflorus* [29] of *C. ladanifer* from Morocco collected in Chefchaounem and Tanger (north of Morocco) had distinct main compounds; nevertheless, this difference could not only be attributed to the different varieties because the harvesting places and extraction processes were different (Table 1). The lowest percentages of α-pinene and camphene in the var. *maculatus* can be attributed to the first extraction process (Soxhlet) using hexane. The monoterpene hydrocarbons, α-pinene and camphene, must have remained in the hexane. In other work, samples of *C. ladanifer* oil obtained from plants of Chefchaounem had, as a main component, 1,8-cineole [30]. In this case, it is not known which variety was analyzed.

The chemical compositions of the resinoid, concrete, and absolute were also assessed by Greche et al. [29] from *C. ladanifer* of Tanger (Table 1). Labdane-type diterpenes were constituents present in these products, as expected.

Along with Tanger, the samples collected in Taza [33] and El Harcha forest (province of Khemisset) [34] produced EOs with viridiflorol as the main compound (Table 1). Camphene at high relative percentages was found in samples of *C. ladanifer* from Oulmes (province of Khemisset) [32] and Tafoughalt [31]. The EO obtained from plants *C. ladanifer* var. *maculatus* of Oulmes presented the diterpene verticiol with the same percentage as that of camphene. Samples from the same province, Khemisset, had diverse chemical profiles (Table 1).

γ-Terpinene and linderol were the most representative compounds of *C. ladanifer* EO from Aknoul (Fès-Meknès region) [35]. Linderol A, a C_26_ compound (C_26_H_30_O_5_), was isolated for the first time in the bark of *Lindera umbellata* [65]. The presence of this unexpected compound can be attributed to the type of extraction process used (microwave-assisted hydro-distillation) or a mistake, because the molecular formula provided by the authors was C_10_H_18_O, not compatible with linderol. Linderol could also be considered a synonym for borneol, but, in the same EO, the authors cite the presence of borneol.

### 3.3. Chemical Composition of Cistus ladanifer EOs from France

In a 1962 review, Martin [66] compared the chemical composition of the EO of *C. ladanifer* from France (Estérel region) and Spain. In both, there were borneol. Acetophenone, benzoic aldehyde, 2,2,6-trimethylcyclohexanone, eugenol, free carboxylic acids, unidentified alcohols, and a mix of terpenes were detected in *C. ladanifer* from France, whereas, in that from Spain, there were 2,3-butadione, furfural, 1,8-cineole, phenols, and α-pinene, but free carboxylic acids were absent. According to the same review, the author claimed that fresh and dried French plants differed in their EO chemical compositions.

In France, α-pinene dominated in almost all three samples of *C. ladanifer* EOs [24,25,26] (Table 1). Previously, Peyron and Alessandri [67] had already reported, in a review work, the presence of α-pinene as a main component in the volatile mixtures of some *C. ladanifer* plants cultivated in Corse [67].

**Figure 9:**
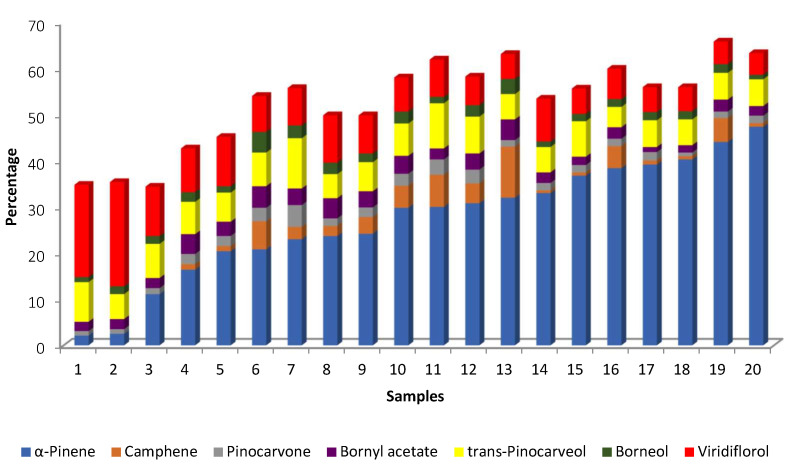
Main compounds (>4.5%) of EOs obtained from 20 populations of *C. ladanifer* cultivated in Corse [24].

The analysis of the EOs of twenty plant populations of *C. ladanifer* cultivated in Corse revealed that there were two samples (samples 1 and 2) in which viridiflorol dominated, with very low amounts of α-pinene (2 and 2.5%) [24] (Table 1). In contrast, samples 19 and 20 had the highest percentages of α-pinene (44.1 and 47.4%, respectively) and lower amounts of viridiflorol (4.9 and 4.7%, respectively) (Figure 9). In addition to these two groups that are easily distinguished, there are two other groups. For example, in samples 3–9, α-pinene ranged from 11.1 to 24.2%, and viridiflorol ranged from 10.7 to 7.8% (Figure 9). In the third group (samples 10–18), the percentages of α-pinene are ≥30% up to 40% and the percentages of viridiflorol ranged from 8.1 to 5.2% (Figure 9).

**Figure 10:**
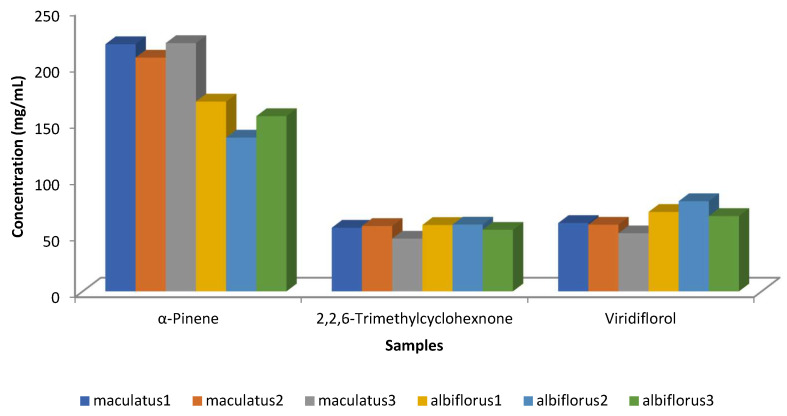
Main compounds (>50 mg/mL) of the EOs isolated from *C. ladanifer* var. *maculatus* and var. *albiflorus* in their locations within the Massif de l’Estérel (south of France) [25].

**Figure 11:**
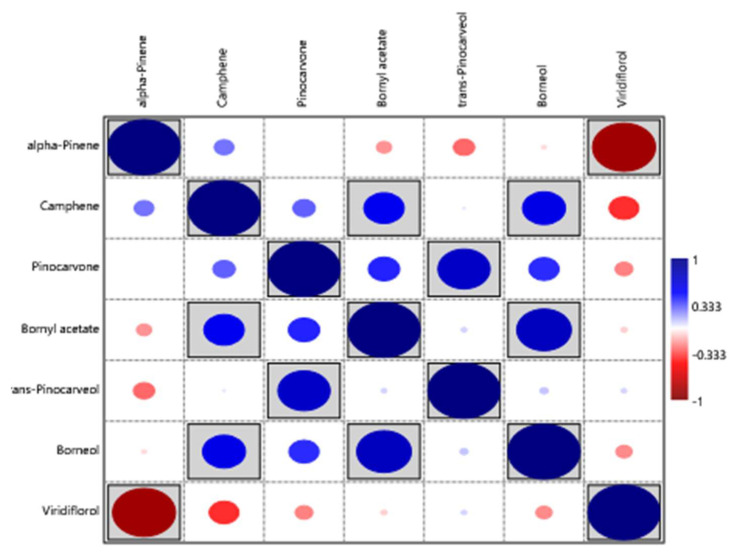
Correlation between the main compounds (>50 mg/mL) of *C. ladanifer* EOs isolated from the var. *maculatus* and var. *albiflorus* in their locations within the Massif de l’Estérel (south of France) [24,54]. Grey background indicates that differences are significant (*p* < 0.05)

Robles et al. [25] examined the chemical composition of the two *C. ladanifer* varieties (*maculatus* and *albiflorus*) in three locations within the Massif de l’Estérel (south of France) (Figure 10). Only concentrations higher than 50 mg/mL were considered in this Figure, and only three compounds (viridiflorol, 2,2,6-trimethylcyclohexanone, and α-pinene) had concentrations above 50 mg/mL. The main component in all samples was α-pinene; nevertheless, the variety *albiflorus* had lower amounts than the variety of *maculatus*. The trend of viridiflorol was the opposite of that of α-pinene (Figure 10). The inverse correlation between α-pinene and viridiflorol, Figure 11, was also observed in the previous works [24,54].

A resinous archeological sample from a tomb in Carthago (6th–5th century BC), housed in the Fragonard Museum (Grasse, France), was identified as labdanum, resin of the *Cistus* species, although no direct documentation supported this identification [68].

However, knowing through ancient documentation that populations in the Mediterranean area used *Cistus* spp, among other species, the authors [68] compared the chemical composition of a commercial cistus EO with that of the archeological sample. The old sample contained very few low-molecular-weight molecules, whereas the EO nearly entirely contains volatile chemicals known as mono- and sesquiterpenes. This suggests that it may be better to look for di- and triterpenes rather than mono- or sesquiterpenes in samples with archeologic origins [68].

### 3.4. Chemical Composition of Cistus ladanifer EOs from India and Germany

The commercial EO from India with the trade name “labdanum essential oil” had, as a main component, α-asarone [27], that is, completely different from the aforementioned *C. ladanifer* EOs. Also, from commercial origin of unknown country, camphene was the main compound followed by α-pinene [58] (Table 1). These findings indicate that some commercially available products labeled as *Cistus ladanifer* oil may have markedly different chemical profiles, raising concerns about authenticity or possible adulteration.

The EO of *C. ladanifer* leaves from the Botanical Institute of the University of Köln had more than 70 compounds present in low proportions of maximal 6.8% [69], in contrast to that verified later by Gülz et al. [70] that reported, as the main compounds of the EO, α-pinene (43%), camphene (12%), and δ-cadinene (7%). These compounds were detected in a fraction obtained from silica gel column chromatography by elution with pentane. In the former work, the authors observed fluctuations in the levels of the compounds over the year [70]. For example, alcohol decreased from spring to autumn, while hydrocarbons and carbonyl compounds increased during the same period. Some compounds reported by Königs and Gülz [69] in the EO extracted from the leaves of *C. ladanifer* include α-pinene, camphene β-pinene, sabinene, myrcene, α-phellandrene, α-terpinene, limonene, γ-terpinene, and *p*-cymene (hydrocarbons); 1,8-cineole (bicyclic ether); 2,2,6-trimethylcyclohexanone, fenchone, α-thujone, *iso*-menthone, benzaldehyde, bornyl acetate, acetophenone, *cis*-citral, *trans*-citral, and geranyl acetate (carbonyl compounds); as well as *cis*-hexen-3-ol-1, *trans*-hexen-3-ol-1, linalool, terpinen-4-ol, borneol, α-terpineol, nerol, geraniol, and eugenol (alcohols) [69].

The phenylpropanoic acid esters with monoterpene alcohols (geranyl phenylpropanoate) were for the first time reported by Proksch et al. [71] in the EO obtained by steam distillation of *C. ladanifer* cultivated in the field of the Botanical Institute of the University of Köln. These compounds were isolated by fractionation through SiO_2_-column and thin layer chromatography. The other compounds identified by these authors were 3-phenylpropanoic acid methyl ester, 2-phenyl-ethanol-1, 3-phenyl-propanol-1, geraniol, and dehydrogeraniol. In the same year, six other compounds (benzyl benzoate, *cis*-ocimene, 2-hydroxy-6-methylacetophenone, pinocarvone, campholene aldehyde, and tagetone) were also identified by the same authors [72]. These compounds were identified by techniques of gas-chromatography coupled with mass spectrometry (GC-MS), infrared spectroscopy (IR), and proton-nuclear magnetic resonance (^1^H-NMR). By reversed-phase high-performance liquid chromatography (RP-HPLC) using octyl and octadecyl silane (C_8_ and C_18_, respectively) bonded silica, water–acetonitrile mixtures as eluents, and ultraviolet (UV) detection at 200, 220, and 254 nm, Strack et al. [73] separated sesquiterpenes and oxygenated monoterpenes. The compounds were separated, and identified by this method, from *C. ladanifer* EO, were benzaldehyde, acetophenone, menthone, 1,8-cineole, fenchone, neral, geranial, bornyl acetate, geranyl acetate, phenylethyl phenylpropanoate, dehydrogeranyl phenylpropanoate, and geranyl phenylpropanoate. Despite this finding, further studies on the EO of *C. ladanifer* preferentially used GC-MS for the separation and identification of its compounds.

## 4. Biological Properties of *Cistus ladanifer* EOs or Volatiles

In addition to the chemical composition of *C. ladanifer* EOs and volatiles summarized in Table 1, numerous studies have reported their biological properties. These include antimicrobial, antioxidant activities, anti-inflammatory, cytotoxicity, and herbicidal effects, among others. The following sections provide an overview of these bioactivities as described by various authors.

### 4.1. Biological Properties of Cistus ladanifer EOs Without Chemical Composition

As aforementioned, the biological properties of *C. ladanifer* EOs were also evaluated by diverse authors even without the chemical profiles of the corresponding EOs provided in very few cases. This section is focused on the diverse biological properties of *C. ladanifer* EOs, in which the respective composition was not provided. For instance, Barbosa et al. [74] evaluated the nematicidal activity, against *Bursaphelenchus xylophilus*, of *C. ladanifer* EO obtained by distillation–extraction (D.-E.) using a Likens-Nickerson apparatus, but the chemical composition was not described. For the extraction of the volatiles, the aerial parts from the vegetative phase and collected in Portugal (Faro) were used. The percentage of inhibition was very low (2.49%). The best activities were found for the EO of *Cymbopogon citratus* (DC) Stapf. (98.12%) and *Mentha pulegium* L. (68.60%). Guimarães et al. [75] studied the antioxidant activity of *C. ladanifer* EO obtained by hydro-distillation from the fresh leaves collected in Portugal (Natural Park of Montesinho, Northeastern Portugal). The EO yield was 0.63%. The antioxidant activities were performed through three distinct methods and, in all cases, the antioxidant activity of *C. ladanifer* EO was higher than the remaining samples (*Citrus latifolia*, *Cupressus lusitanica,* and *Eucalyptus gunnii*). The methods used were the capacity for scavenging the 2,2-diphenyl-1-picrylhydrazyl free radicals (DPPH), reducing power, and inhibition of β-carotene bleaching that determined the capacity to inhibit lipid peroxidation. The results were expressed as concentrations, providing 50% of radical scavenging activity (EC_50_). The EC_50_ found for the DPPH, reducing power and inhibition of lipid peroxidation were 36.28, 4.00, and 0.12 mg/mL, respectively.

Kotba et al. [76] studied the effect of *Thymus vulgaris*, *Origanum compactum*, *Rosmarinus officinalis*, *Eucalyptus* sp., *Salvia* sp., *Lavandula stoechas,* and *Cistus ladanifer* EOs on the growth of *Rhizoctonia solani* strains, isolated from many crops in Morocco based on radial mycelial growth on potato dextrose agar at different pH and temperature levels. The EO obtained from the leaves of *C. ladanifer* showed the lowest level of growth inhibition rates on most of *R. solani* isolates, in contrast to those of *O. compactum* and *T. vulgaris*.

Biopesticides, including EOs, have a history of use as potato sprout suppressants. When potatoes are harvested, the many buds on their surfaces are in a natural state of dormancy and will not sprout. This natural dormancy is temporary, and the buds will start to sprout after several months of storage. Limiting potato sprouting during storage is necessary because it reduces tuber weight, alters the texture and nutritional content of the tuber, and produces the poisonous alkaloid solanine. Thoma et al. [77] studied the sprout suppressant effect of twenty-one EOs in cv. Ranger Russet potatoes at room-temperature storage. For shorter storage periods, the EOs of *C. ladanifer*, *Ocimum basilicum*, *Ormenis mixta*, and *Salvia sclarea* greatly decreased the length of the sprouts, while those of *Laurus nobilis* and *Cinnamomum zeylanicum* (bark) significantly decreased the number of sprouts. The chemical composition of *C. ladanifer* was not described; only that of *Artemisia herba-alba* was provided because this EO was the most effective at suppressing both sprout length and sprout number over 90 days.

### 4.2. Antioxidant Activity

Antioxidant activity of *C. ladanifer* EOs was determined by several methods (ferric reducing antioxidant activity power (FRAP), β-carotene bleaching assay, reducing power activity, oxygen radical absorbance capacity (ORAC), chelating activity, and the ability to scavenge DPPH and 2,2′-azino-bis(3-ethylbenzothiazoline-6-sulfonic acid) (ABTS) free radicals). The results were presented in different ways as can be seen in Table 1. The most used methods were those of DPPH and ABTS. The *C. ladanifer* EO from commercial origin of India possessed the ability for scavenging DPPH and ABTS free radicals with IC_50_ values of 7.3 and 1.13 μL/mL, respectively [27]. An EO of *C. ladanifer* from Morocco also showed ability for scavenging those free radicals but with IC_50_ values of 178.29 and 134.02 μg/mL for DPPH and ABTS, respectively, which is much better than that from India. The capacity for scavenging DPPH was also determined for Portuguese *C. ladanifer* EOs (IC_50_ between 0.691 and 0.9% (*v*/*v*) (Table 1). According to Oliveira et al. [17], such values can be considered as poor due to the low antioxidant activity index (0.0037). These authors also evaluated the antioxidant activity of the hydrolates and concluded that the activity (IC_50_ value = 43%, *v*/*v*) was much lower than that observed for the corresponding EO as expected, given the reduced levels of volatiles in the hydrolates. The capacity of the EOs of rockrose to scavenge DPPH free radicals were determined by other teams, but the results were not presented through IC_50_ values but by percentages of inhibition or Trolox equivalent/g, making it difficult to compare the results (Table 1).

The relationship between antioxidant activity and EO composition throughout the year was evaluated [54]. Regarding the correlation between ORAC values and EO composition, significant correlations in the EOs of the 7-year-old population were found for contents of verbenene, which demonstrated a strong negative correlation, and ledene that showed a moderate positive correlation. In the EOs of the older population (12 years old), negative correlations were found for contents of verbenene, myrtenal, and borneol; and positive correlations were found for limonene and α-pinene. Mediavilla et al. [51] found higher antioxidant activity of the *C. ladanifer* EOs, as measured by the ORAC assay, in October than in September, such as previously reported [54], although with lower differences between the harvesting periods than those reported [54]. However, there are authors [42] who did not find any correlation between antioxidant activity, measured through the ABTS method, and different harvesting periods of the year. This variability is not uncommon, and even occurs very frequently with other plants, because it seems to be related to the chemical composition of the EOs, which despite having a similar profile, the has main compounds that are present in different proportions [78]. Furthermore, EOs’ antioxidant properties can be ascribed to both their major molecules and their minor constituents, which may have antagonistic or synergistic effects [79].

The antioxidant activities of *C. ladanifer* EOs at 2 mg/mL, from Andévalo and Cerezal (Spain) with α-pinene and viridiflorol as main compounds, respectively, were also assessed using cellular murine macrophage cell line RAW 264.7 using azobis(2-methylpropionamidine) dihydrochloride (AAPH) and 2,7-dichlorofluorescein diacetate (DCFHDA) as a fluorescent marker. Both EOs had similar percentages of inhibition (83.24 and 81.13%, respectively) (Table 1) [49].

### 4.3. Antimicrobial Activity

Diverse microorganisms were tested, and the response differed as expected, not only due to the characteristic response of the microorganism to the natural product but also due to the chemical variability of the EOs. Diverse Gram-positive (*Staphylococcus aureus*, *S. epidermidis*, *Listeria innocua*, *L. monocytogenes*, *Bacillus subtilis*, *B. cereus*, *Enterococcus faecalis*, *Cutibacterium acnes*) and Gram-negative microorganisms (*Escherichia coli*, *Campylobacter jejuni*, *Pseudomonas aeruginosa*, *Pseudomonas savastanoi*, *Salmonella typhy*, *Acinetobacter baumanni*, *Proteus mirabilis*, *Klebsiella pnuemoniae*, *Morganella morganii*) were used. Some of the microorganisms were repeatedly used by several teams. As shown in Table 1, the effect against yeasts and fungi was also tested.

The most researched Gram-positive microorganism for *C. ladanifer* EOs was *Staphylococcus aureus*. There was activity in all studies, with inhibition zones measuring between 11 and 23 mm. Compared with the reference strain (11 mm), the EO containing 36% α-pinene was more efficient against methicillin-resistant *Staphylococcus aureus* (MRSA) (23 mm) [40]. The minimum inhibitory concentration (MIC) value found was 10 µL/mL in the Portugal and Morocco EOs and 16 µL/mL in the Portugal sample. Only the Moroccan EO had violiflorol as its primary constituent; other EOs had α-pinene (Table 1).

The anti-*Listeria monocytogenes* effect of the *C. ladanifer* EO revealed that Cerezal (Spain) EOs with more viridiflorol (24%) and lower α-pinene (19%) had lower MIC values (0.3 mg/mL) than Andévalo samples (0.6 mg/mL) [49]. Viridiflorol may be more successful than α-pinene, as evidenced by a similar pattern seen for *Enterococcus faecalis*. The activity against *E. coli*, however, was identical for both samples (0.6 mg/mL). MIC values for *Listeria* and *E. faecalis* in a sample from Mértola, Portugal, were 8 µL/mL [41], which were comparable to the Andévalo sample that was high in α-pinene. For *E. faecalis*, the Portuguese EO had a lower MIC (Table 1).

The impact of α-pinene-rich *C. ladanifer* EO on *Cutibacterium acnes* strains from several phylotypes that are linked to acne and healthy skin was studied [17,44]. MIC values varied with phylotype, ranging from 0.06% to 0.5% (*v*/*v*) (Table 1). Additionally, the EO inhibited the formation of biofilms; the half maximum effective concentration (EC_50_) values for biofilm biomass ranged from 0.06% to >0.5% (*v*/*v*). Nevertheless, *Thymus x citriodorus* EO, which mainly constitutes thymol, 1,8-cineole, and geraniol, show greater activity than *C. ladanifer* EO [44].

A study made by Chaloupková et al. [54], in which the annual variation in yield, chemical composition, and antibacterial activity against *B. cereus* of rockrose EO was evaluated in young plants (7 years old) and older ones (12 years old), permitted the conclusion that the older ones presented better activities than the youngest ones, although no correlation had been found between MIC values and EO composition. In the same work, the results of vapor phases referred to as agar (lid) showed weaker antibacterial activity compared with the liquid phase (broth) [54].

*Escherichia coli* and *Pseudomonas aeruginosa* were the most often employed species among Gram-negative bacteria to investigate the antibacterial activity of *C. ladanifer* EOs (Table 1). In all cases and regardless of the dominant compounds of the EOS, they possessed an antimicrobial effect. There are significant differences between the results that have the same unities and that are easy to compare. Regarding *E. coli*, a MIC value of 25 mg/mL was found for an EO of *C. ladanifer* from Morocco, and 0.6 mg/mL for an EO of Spain. This disparity may have been partially caused by the chemical compositions of the EOs, which differed significantly between Moroccan EO (camphene and verticiol) and Spanish EOs (α-pinene or viridiflorol) (Table 1). However, since the same EO of *C. ladanifer* inhibited the growth of *E. coli* SA/4 (an extended spectrum beta-lactamase producer) with an experimental inhibition zone of 20 mm, while the inhibition zone for the reference strain *E. coli* ATCC 25922 was smaller (10 mm) [40], other factors might affect the response.

α-Pinene and viridiflorol + α-pinene as main compounds of Spanish EOs of *C. laddanifer* collected in different places were able to prevent the growth of *P. aeruginosa*, *Klebsiella pneumoniae*, and *Proteus mirabilis* in the same way as they presented equal MIC values (2.5 mg/mL, >2.5 mg/mL, and >2.5 mg/mL, respectively) [49]. The *C. ladanifer* EO with viridiflorol + α-pinene had better activity against *Morganella morganii* (MIC = 0.6 mg/mL) than the EO in which α-pinene dominated (Table 1) [49].

Regardless of their chemical compositions, *C. ladanifer* EOs demonstrated the capacity to inhibit the growth of yeasts and fungi, albeit at varying intensities. When compared with the other *C. ladanifer* EOs, one EO had the lowest MIC values for *Aspergillus niger*, *Tricophyton rubrum*, and *Candida albicans* (0.001, 0.01, and 0.001 mg/mL, respectively), making it the most effective EO against those microorganisms. The MIC values differed from the others in a significant way. The main compounds in the EO were verticiol and camphene (Table 1) [32].

By assessing the impact on ergosterol biosynthesis, the mechanism of action of *C. ladanifer* EO against aflatoxigenic *Aspergillus flavus* AF-M-K5 was investigated [27]. Ergosterol content dramatically dropped as EO concentration rose; inhibition percentages ranged from 4% at 0.1 μL/mL to 84.52% at 0.5 μL/mL (Table 1). This inhibition implies that the fungal cell membrane was affected mainly by α-asarone, the main compound of the EO. Furthermore, *C. ladanifer* EO totally stopped *A. flavus* from producing aflatoxin B1 at 0.5 μL/mL [27].

Brindhadevi et al. [80] evaluated the antimicrobial activity of silver nanoparticles functionalized with labdanum oil. These functionalized nanoparticles were used to produce wound dressings to enhance the healing wounds. An increased antibacterial activity was observed when the produced wound dressings and silver nanoparticles were examined for antimicrobial activity. The data revealed that at the end of 24 h incubation at 37 °C, silver nanoparticles and silver nanoparticles functionalized with *C. ladanifer* oil exhibited inhibitory actions against *Klebsiella pneumoniae*, with inhibition zone diameters of 37 mm and 42 mm, respectively. Silver nanoparticles and silver nanoparticles functionalized with *C. ladanifer* oil had inhibitory zones of 40 and 41 mm for *A. niger*, 35 and 45 mm for *S. aureus*, 34 and 35 mm for *B. subtilis*, and 40 and 42 mm for *E. coli*, respectively. The chemical composition was not assessed, therefore it was difficult to attribute some of these abilities to some compounds, although the authors had discussed their results based on the chemical composition of the oil from other oil samples [80].

### 4.4. Phytotoxical Activity

The reduced plant diversity and richness in the presence of *C. ladanifer* is believed to be due to the allelochemicals released by its leaves and stems. These compounds remain in the soil for a long time, breaking down very slowly in the soil. Despite having low concentrations (of the order of μg/g soil), aglycone flavonoids (apigenin, 3-*O*-methyl kaempferol, 4’-*O*-methylapigenin, 7-*O*-methylapigenin, 3,4’-di-*O*-methylkaempferol and 3,7-di-*O*-methylkaempferol) remaining on the soil for long periods may change its properties and inhibit the germination and growth of other species, explaining the phytotoxic activity attributed to *C. ladanifer* [81,82,83,84]. Diterpenes are also responsible for the phytotoxicity, and three were identified in *C. ladanifer* exudates (oxocativic acid, 7-oxo-8-labden-15-oic acid, and 6β-acetoxi-7-oxo-8-labden-15-oic acid) [15,85]. The allelopathic potential of *C. ladanifer* secondary metabolites, conjointly and not separately, is also dependent on the environmental factors, high temperatures, and long photoperiods. These three factors increase the phytotoxicity in inhibiting both germination and seedling development [16,86].

**Figure 12:**
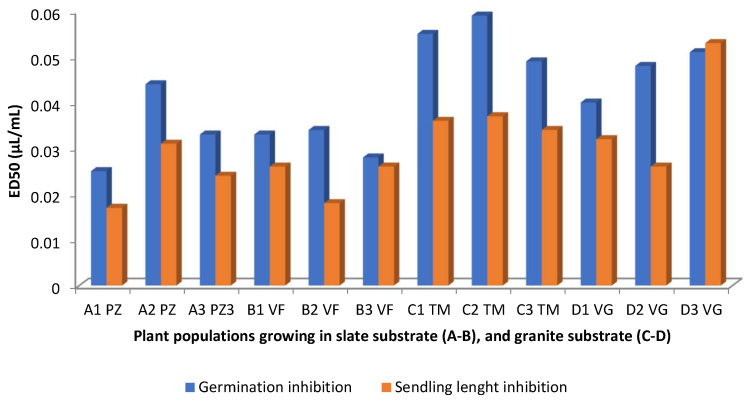
Germination and seedling length inhibition of *Raphanus sativus* by *C. ladanifer* EOs obtained from plant populations (A1,B1,C1,D1; A2,B2,C2,D2; and A3,B3,C3,D3 growing in PZ: Pozuelo de Zarzón (slate substrate); VF: Valverde del Fresno (slate substrate); TM: Torre de Don Miguel (granite substrate); VG: VillasBuenas de Gata (granite substrate) [53].

With very few exceptions, diterpenes are absent in the EOs of *C. ladanifer*, at least at concentrations >4.5% (Table 1), with monoterpenes, sesquiterpenes and other groups of compounds being the ones that are present in those EOs at relatively high concentrations. However, there are works showing that sesquiterpene-rich EOs present phytotoxic activity [87]. In this way, some authors [53] evaluated the effect of viridiflorol-rich EO of *C. ladanifer* from different locations in Spain on the seed germination and seedling length inhibition of *Raphanus sativus* (Figure 12 and Table 1). Both seed germination and seedling length were mostly affected by the EOs coming from the slate substrate zone, because lower ED_50_ meant higher inhibition. Comparing this Figure with that of Figure 7, it is possible to conclude that there is a negative correlation between the percentages of viridiflorol and ED_50_ values and a positive correlation between the percentages of α-pinene and the ED_50_ values. The authors [53] also found a negative correlation between these values and the percentages of the diterpene 15-nor-labdan-8-ol, not represented in Figure 7 or in Table 1 because the percentages were <4.5%. *C. ladanifer* EOs with high percentages of viridiflorol from Morocco also have the capacity to inhibit the germination of tomato seeds [33]. *Raphanus sativus* seed germination and seedling length were likewise inhibited by the *C. ladanifer* EO, which contained the sesquiterpene ledol at a rather high concentration (19%) [52], indicating that oxygenated sesquiterpenes had an inhibitory effect on weed seed germination [53]. Still using a Spanish rockrose EO, in which *trans*-pinocarveol (20%) and viridiflorol (14%) were main compounds, it was possible to particularly inhibit the seed germination of *Amaranthus hybridus*, *Conyza canadensis*, and *Parietaria judaica*, regardless of the concentrations of the EOs used (0.125 to 1 µL/mL) [46].

**Figure 13:**
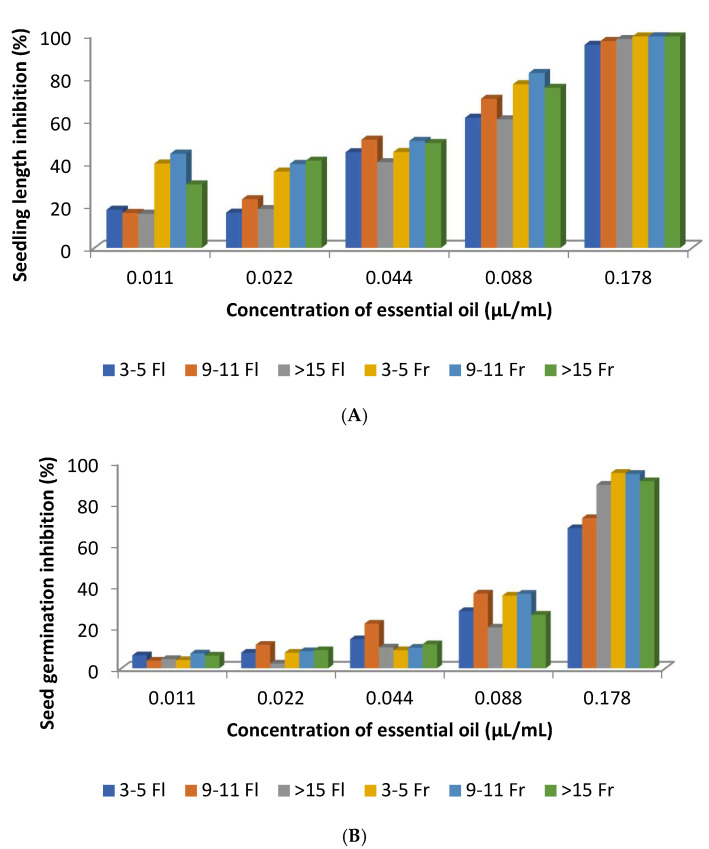
Seedling length inhibition (**A**) and seed germination inhibition (**B**) by *C. ladanifer* EOs at different concentrations obtained from different aged plant at two phenological stages in Extremadura (Spain). 3–5 Fl: plants of 3–5 years old at flowering phase; 9–11 Fl: plants of 9–11 years old at flowering phase; >15 Fl: plants of >15 years old at flowering phase; 3–5 Fr: plants of 3–5 years old at fruit maturation phase; 9–11 Fr: plants of 9–11 years old at fruit maturation phase; >15 Fr: plants of >15 years old at fruit maturation phase [55].

With the exception of the concentration 0.44 µL/mL, the EOs obtained during the fruit maturation stage exhibited stronger inhibition of *Raphanus sativus* seedling length compared with those collected during flowering (Figure 13A). This indicates that the phytotoxic activity of *C. ladanifer* EOs varies according to the plant’s phenological stage. In contrast, this trend was less evident in seed germination inhibition (Figure 13B). When comparing these Figures to Figure 8 it seemed that higher percentages of α-pinene and lower concentrations of 2,2,6-trimethylcyclohexanone in the EOS obtained from plants at fruit maturation better inhibited the seedling length of *R. sativus* (Figure 8 and Figure 13A,B). This can be considered for compounds that are present in the *C. ladanifer* EOs at concentrations higher than 4.5%. Other correlations were made by the authors [55] between some other minor constituents of the EOs and the seedling length of *R. sativus*. For example, EO samples from the fruit maturation stage that are richer in monoterpenes and sesquiterpenes have higher phytotoxic activity on the early growth of *R. sativus* seedlings than the EO samples from the flowering stage, which have a higher percentage of diterpenes such as 15-nor-labdan-8-ol [55]. This is somehow distinct from what was previously reported [53]. Regarding the capacity to inhibit seed germination, there is no correlation between the phenological stage of the plant and phytotoxicity activity as found for the seedling growth, suggesting diverse inhibitory mechanisms involved in the activities. The highest percentages of monoterpene hydrocarbons in October than in August were also reported in other work [56]. In contrast to the aforementioned results, some other authors [27] demonstrated that the *C. ladanifer* EO with 79% of α-asarone did not influence seed germination and length of the radical and plumule of seedings of *Brassica juncea*, *Arachis hypogaea*, *Sesamum indicum*, *Helianthus annuus*, and *B. campestris*.

### 4.5. Citotoxicity, Anti-Inflammatory, and Other Biological Activities

In vitro cytotoxicity of *C. ladanifer* EOs was evaluated using several cell lines (Table 1). Ecotoxicity was also checked through the determination of the acute toxicity performed through the model organism *Daphnia magna*. The result showed that the EO in which α-pinene dominated [39] was not toxic because the EC_50_ (concentration to immobilize 50 per cent of the *D. magna* after 48 h) was higher than 100 mg/L. The EC_50_ value was 199.7 mg/L.

The estimated half-maximal inhibitory concentrations for cellular viability (IC_50_) obtained for the two tested cell lines (mouse fibroblasts L929 and macrophages RAW264.7) and estimated half-maximal effective concentrations for nitric oxide (NO) production in macrophages (EC_50_) were 0.027, 0.012, and 0.002% (*v*/*v*) (Table 1) [17] for the α-pinene-rich EO of *C. ladanifer* from Portugal. The same assays were made with the corresponding hydrolates, and the values were significantly higher (Table 2). According to the authors [17], the cytotoxicity on L929 fibroblasts was followed to determine wound-healing potential, and the results showed that the hydrolates were more biocompatible than the EO due to the higher IC_50_ values. The anti-inflammatory activity of *C. ladanifer* EO measured through the NO production in macrophages after being exposed to lipopolysaccharide (LPS) at 1 µg/mL was also better than the respective hydrolates (lower EC_50_ values, higher activity) (Table 1 and Table 2).

The in vitro cytotoxic activity on human cancer cell lines (breast adenocarcinoma (MCF7, T47D, and MDA-MB-231), chronic myelogenous erythroleukemia (K562), and neuroblastoma cell lines (SH-SY5Y) of sixteen commercial EOs were evaluated [32], including that of *Cistus ladanifer*. The IC_50_ values in parts-per million (ppm) are depicted in Table 1. The K562 cells were the most sensitive to the *C. ladanifer* EO since they presented the lowest IC_50_ value (Table 1). However, the *Cinnamomum zeylanicum* and *Litsea cubeba* EOs were the most active with IC_50_ values <11.5 ppm, whereas for *C. ladanifer* EO, the IC_50_ value was >45 ppm. (*E*)-Cinnamaldehyde, and citral were the major compounds in *C. zeylanicum* and *L. cubeba* EOs, whereas α-pinene dominated the *C. ladanifer* EO [48]. In another study [49], *C. ladanifer* EO showed high potential on lung carcinoma NCl-H460 since presenting the concentration of the sample caused 50% of cell growth inhibition (GI_50_), a value lower (14.27 µg/mL) than those of the remaining tumor cell lines tested, particularly for the sample collected in Andévalo (Spain), in which α-pinene was the main component (Table 1). All of the samples in the non-tumor cell lines also showed cytotoxicity; nevertheless, with GI_50_ values higher than the tumor cell lines. These results might indicate that these *C. ladanifer* EOs can be used in some circumstances without being harmful [49].

The anti-inflammatory activity of *C. ladanifer* EOs from Spain (Andévalo and Cerezal) was also assayed [49] through the evaluation of the inhibition of the LPS-induced NO production on RAW264.7. The IC_50_ values were 19.27 and 21.00 µg/mL, respectively, that is, without significant difference between the EOS collected in both places.

The inhibitory activity of some enzymes (α-amylase, α-glucosidase) were also evaluated in *C. ladanifer* EOs from Morocco [32] and of commercial origin [58], with distinct chemical compositions (Table 1); nevertheless, the results could not be compared since the ways of presenting them were different. The inhibitory activities of tyrosinase, acetylcholinesterase, and butirylcholinesterase were also evaluated [58], as well as that of pancreatic lipase [32]. The inhibitory activities of the enzymes α-amylase, α-glucosidase, acetylcholinesterase, butirylcholinesterase, and tyrosinase, potently associated with diabetes, Alzheimer’s disease, and pigmentation disorders, respectively, were attributed to the presence of camphene, α-pinene, and bornyl acetate, the main compounds of the commercial EO of *C. ladanifer* [58].

## 5. By-Products Obtained from *Cistus ladanifer* EOs Distilleries

During the research for the current review on *Cistus ladanifer* EOs, some articles that did not focus on EOs were found. Rather, the authors focused on the use of the plant material that resulted from steam distillation as well as hydrolates. Therefore, a brief review of this topic was also deemed pertinent.

The residual material obtained after steam distillation of the aerial parts of *C. ladanifer* is usually used as firewood or fuel for further hydro-distillations after drying at room temperature (usually 1 day). These solid materials from *C. ladanifer* distilleries are lignocellulosic materials, which can be an income to produce biofuels and bioproducts. Alves-Ferreira et al. [88,89] investigated the utilization of mild autohydrolysis processes to selectively hydrolyze hemicelluloses from *C. ladanifer* residual material, producing hemicellulose-derived oligosaccharides and extracting water-soluble phenolic compounds (4-methylguaiacol, 4-ethylguaiacol, 4-vinylguaiacol, syringol, 4-methylsyringol, 4-vinylsyringol, *trans*-4-propenylsyringol, *trans*-coniferaldehyde) targeted to potential added-value applications and cellulose/lignin-enriched solids. The same authors also concluded in other research that the distillery residues with residues high concentrations of extractives, particularly polar extractives that were rich in phenolics (syringol derivatives such as 4-methylsyringol, 4-ethylsyringol, 4-vinyl syringol, 4-allylsyringol, *cis*-4-propenylsyringol, *trans*-4-propenyldyringol, syringaldehyde, homosyringaldehyde, acetosyringone, syringylacetone, and *trans*-sinapaldehyde; guaiacol derivatives such as 4-methylguaiacol, 4-ethylguaiacol, 4-vinylguaiacol, eugenol, *trans*-isoeugenol, vanillin, 1-(4-hydroxy-3-methoxyphenyl)-propyne, homovanillin, acetoguaiacone, guaiacylacetone, trans-coniferyl alcohol, and trans-coniferaldehyde; as well as p-hydroxyphenyl derivatives such as phenol, *o*-cresol, *p*-cresol, *m*-cresol, 2,3-dihydrobenzofuran, and 2,3-dimethyl-phenol), flavonoids (apigenin, isoquercetin, gallocatechins), and tannins (particularly in the stem extractives) were found, which demonstrated strong antioxidant activity, being a significant chemical characteristic of *C. ladanifer* compounds and their distillery residues [90]. Later on, Alves-Ferreira et al. [91] studied the delignification of extracted and hydrothermally pretreated biomass using two organosolv processes, ethanol/water mixtures, and alkali-catalyzed glycerol and by an alkali (sodium hydroxide) process under different reaction conditions, followed by the evaluation of the phenolic composition of soluble lignin, determined by capillary zone electrophoresis and pyrolysis-GC-MS. Low-molecular-weight phenolic compounds were identified in all the delignification liquors. Previously, Tavares et al. [92] also obtained phenolic-rich extracts from the remaining extracted solid residues of *C. ladanifer* using ultrasound-assisted extraction with ethanol (salicylic acid, 3-hydroxybenzoic acid, apigenin, quercetin, syringic acid) and 70% acetone (salicylic acid, 3-hydroxybenzoic acid, apigenin, gallocatechins, syringic acid). The waste distilled by-products remaining after steam distillation of the underutilized biomass from *Cistus ladanifer* can also be a natural source of other high value products with biological activities, namely, phenolic compounds (quercetins, gallocatechins, hydroxycinnamic acids derivatives, gallic acid), which were also studied by Tavares et al. [92]. Some of these compounds (gallic acid, ellagic acid, apigenin, kaempferol diglycoside, kaempferol methyl ether, kaempferol dimethylether) were also previously described by Sánchez-Vioque et al. [93] in *C. ladanifer* solid residues after steam distillation to obtain EOs. The extracts obtained by Soxhlet or ultrasound-assisted extraction containing those compounds showed significant antioxidant activity. Taking into account all of these findings, using *C. ladanifer* biomass for an extractive lignocellulosic-based biorefinery is a possible way that could raise profits for the current distilleries of essential oils [88].

Additional compounds can be produced from hemicellulose hydrolysates derived from *C. ladanifer* waste gathered from an EO distillery, such as D-lactic acid. *Escherichia coli* JU15 can effectively use both hemicellulose- and cellulose-derived sugars as the carbon source to produce D-lactic acid, with a high yield (92–99%). *E. coli* JU15 can also metabolize glucose and xylose while retaining high lactate yields when moderate concentrations of putative inhibitors, such as furans, formic acid, acetic acid, and phenolic compounds, are present in non-detoxified hydrolysates. Lactic acid is an organic acid that finds widespread use in the chemical, pharmaceutical, cosmetic, and food industries. The demand for polylactic acid, a biodegradable and thermostable semi-crystalline polymer used as a sustainable alternative to plastics made from petrochemicals, has increased dramatically due to its potential applications [94].

Álvaro et al. [95] demonstrated the potential of utilizing solid residues after EO extraction as energy resources. Biogas was produced by direct anaerobic digestion of the post-distillation biomass with a particle size of less than 1 mm and an inoculum obtained from the anaerobic digester facility of the Wastewater Treatment Plant of Soria (Spain). In comparison to the solid-fuel method, *C. ladanifer* exhibited noteworthy biomethane production with a total energy recovery of between 25% and 45%. According to the authors [95], distillation by-products of *C. ladanifer* can be potentially considered as co-substrate for biogas generation with a competitive methane yield, similar to other lignocellulosic wastes.

Hydrolates, hydrosols, or floral water correspond to the distilled water that remains after the hydro- or steam distillation and the separation from the corresponding EO. The hydrolate is usually rich in EO water-soluble compounds. This hydrolate can be considered a by-product that can be obtained within the biorefinery concept from forest biomass. It possesses antimicrobial, antioxidant, anti-inflammatory, antispasmodic, and relaxing properties, among others, beyond the pleasant odor and flavor, interesting for the perfumery, cosmetic, and flavor industries [42].

Table 2 depicts the chemical composition of hydrolates obtained from diverse regions and the corresponding biological properties found by the authors. Generally, the activities are poor or even absent as expected since the amounts of terpenes are lower than those found in the essential oils.

After steam distillation or hydro-distillation, Tavares et al. [42] also recovered the hydrolates and assessed their chemical composition (Table 2 and Figure 6 in Section 3.1). The hydrolates included very small percentages of viridiflorol and bornyl acetate and almost no hydrocarbon monoterpene molecules (tricyclene, α-pinene, camphene, and limonene) (Figure 6). In contrast, all hydrolates had higher percentages of verbenone, terpinen-4-ol, and *trans*-pinocarveol, whereas samples that were obtained by steam distillation had higher percentages of 2,2,6-trimethylcyclohexanone and borneol (Figure 6).

The EOs and hydrolates were also obtained by Pérez-Izquierdo et al. [52] from *C. ladanifer* collected in Spain (Guijo de Granadilla, north of Extremadura) by hydro-distillation. The hydrocarbons were absent in the hydrolate whereas pinocarvone (6.31%) and *trans*-pinocarveol (10.96%) were only present in this product. The percentage of viridiflorol was lower in the hydrolate (3.66%) than in the EO (7.11%) as reported for Portuguese samples (Table 2 and Figure 6 in Section 3.1) [42]. The same pattern was observed for 2,2,6-trimethylcyclohexanone (2.82% in the EO and 10.31% in the hydrolate) as observed in the Portuguese samples, mainly in those obtained by steam distillation. The percentages of bornyl acetate were similar in both the EO (4.80%) and the hydrolate (4.38%) (Table 1 and Table 2). In conclusion, in the two independent assays, there is a similar pattern.

*C. ladanifer* aerial parts collected in the central-west region of Portugal produced an EO rich in α-pinene (35.8%) that was absent in the hydrolate [43], as expected. However, the ketone 2,2,6-trimethylcyclohexanone that was at higher concentrations in hydrolates in the aforementioned works, in this case, was absent in the hydrolate, although it was at 6.7% in the EO. Other differences were also possible to detect: the presence of endo-borneol (8.4%), *p*-cymen-8-ol (10.7%), myrtenol (11.2%), verbenone (9.8%), and 4-hydroxy-3-methylacetophenone (21.6%) in the hydrolate, not reported by the other authors [42,52]. For commercial Portuguese-origin *C. ladanifer* hydrolates (Proentia^®^ company), the lack of monoterpene hydrocarbons was also reported [17], although the EO was high in α-pinene.

## 6. Conclusions

*Cistus ladanifer* L., a widely recognized Mediterranean shrub, is a highly versatile species with significant potential for cosmetic and pharmaceutical applications. The chemical composition of its essential oils and volatiles varies across Mediterranean regions, with α-pinene, camphene, and viridiflorol commonly present, though in differing concentrations depending on geographic origin, season, plant age, storage conditions, and extraction methods.

The biological activities of *C. ladanifer* EOs and hydrolates, particularly their antibacterial, antioxidant, anti-inflammatory, cytotoxic, and phytotoxic properties, highlight the value of this species. To fully exploit its potential, it is crucial to develop improved preservation methods for EOs and hydrolates, enabling broader application in various industries.

Recent research has explored innovative approaches such as nanoencapsulation, bioactive film development, and the use of EOs in food and pharmaceutical packaging [38,96]. Additionally, the valorization of distillation by-products, including hydrolates, lignocellulosic residues, and phenolic-rich extracts, supports the sustainable utilization of *C. ladanifer*. Standardizing extraction processes and promoting the use of underexploited by-products are essential steps toward maximizing the benefits of this valuable yet underutilized species.

Moreover, the lignocellulosic residues left after essential oil extraction represent a valuable resource for biorefinery processes and the recovery of phenolic-rich compounds. Rather than viewing *C. ladanifer* as an invasive or undesirable species, especially in the increasingly arid and fire-prone Mediterranean basin, it should be recognized as a multifunctional natural resource that contributes to sustainable forestry, climate adaptation strategies, and circular bioeconomy initiatives.

## Figures and Tables

**Table 1:** Main compounds present in EO and volatiles of *Cistus ladanifer* and biological properties.

Origin	Plant Part Used	Extraction Type/Identification/Yield	Main Identified Compounds (>5%) ●	Biological Properties	Reference
France Cultivated in France (Corsica) from Spanish plant origin	Leaves and stems	Hydro-distillation/GC-FID, silica gel flash chromatography and ^13^C-NMR/0.07% (bulk sample; from 0.16 to 0.41% fresh material (individual samples)	Bulk sample: α-pinene 39, viridiflorol 12 (identification by ^13^C-NMR and quantification by GC) Individual 20 samples (minimal–maximal): α-pinene 2–47, viridiflorol 5–23, *trans*-pinocarveol 4–11, camphene not detected–11, pinocarvone 1–5, bornyl acetate 1–5, borneol 1–5	Not determined	[24]
France (Massif de l’Estérel, South of France)	Young shoots, leaves, and stems of. *C. ladanifer* var. *maculatus* and *C. ladanifer* var. *albiflorus*	Steam distillation in a modified Dean and Stark apparatus/GC-MS/0.119% (*w*/*w*) fresh material (var. *albiflorus*); 0.081% (*w*/*w*) fresh material (var. *maculatus*)	Mean (μg/μL) > 10: *albiflorus*: α-pinene 153, viridiflorol 72, 2,2,6-trimethylcyclohexanone 58, terpinen-4-ol 35, verbenone 34, *trans*-pinocarveol 33, borneol 27, α-campholenic aldehyde 27, ledol 19, camphene 19, benzaldehyde 14, carveol 14, myrtenol 13, limonene 12, *p*-cymene 12 *maculatus:* α-pinene 216, viridiflorol 57, 2,2,6-trimethylcyclohexanone 54, verbenone 41, terpinen-4-ol 32, *trans*-pinocarveol 31, camphene 28, borneol 27, α-campholenic aldehyde 27, ledol 16, limonene 13, carveol 13, α-terpinene 12, *p*-cymene 11, benzaldehyde 11, myrtenol 11	Not determined	[25]
France (Corse) Commercial from Huiles Essentielles et Hydrolats de Corse	Unknown	GC-MS/not determined	α-pinene 47, globulol 6, camphene 5	**Antimicrobial activity** - MIC % (*v*/*v*) (*Campylobacter jejuni*) 0.125	[26]
India (commercial obtained from Meena Perfumery Kannauj, India)	Labdanum essential oil	GC-MS/not determined	α-asarone * 79, camphene 6	**Antimicrobial activity** - MIC = 0.6 μL/mL *(Aspergillus flavus* strain AF-M-K5 isolated from *Brassica juncea* seeds) - Absolute suppression of aflatoxin B1 production by *A. flavus* AF-M-K5 at 0.5 μL/mL. - Percentage mycelial inhibition at 0.6 μL/mL = 100% for *Alternaria alternata*, *A. humicola*, *A. tenuis*; *Aspergillus chevalieri*, *A. luchuensis*, *A. minutus*, *A. niger*, *A. repens*, *A. terreus*, *A. versicolor*; *Cladospoium cladosporioides*, *C. humicola*; *Curvularia lunata*; *Mucor* sp.; *Peniclium luteum*, *P. purpuroger*; *Rhizopus stolonifer*; *Terbicularia* sp. - Percentage inhibition of ergosterol content at 0.1, 0.2, 0.3, 0.4, 0.5 μL/mL: 4, 16, 19, 28.5, 84,52, respectively. - Decrease in methylglyoxal content with increasing concentration of EO: 0.6 and 1.0 μL/mL = 505.42 and 440.83 μM/g fresh weight. **Antioxidant activity** - IC_50_ = 7.3 μL/mL and 1.13 μL/mL for the DPPH and ABTS methods, respectively. - **Phytotoxicity** 100% germination and without reduction in the length of radical and plumule of seedlings: *Brassica juncea* (var. Varuna and Kranti), *Arachis hypogaea* (var. Chandra and Kaushal), *Sesamum indicum* (var. T-12 and Shekhar), *Helianthus annuus* (var. Modern and Surya), *Brassica campestris* (var. Pusa swarnim) fumigated with EO showed.	[27]
Morocco (Chefchaouen northern Morocco)	Leaves	Extraction with hexane (Soxhlet apparatus)/hydro-distillation/GC-FID/GC-MS/0.18% fresh material weigh	pinocarveol 8, viridiflorol 7, bornyl acetate 6	Not determined	[28]
Morocco (Tanger, North Morocco)	Leaves and small branches	Hydro-distillation/GC-FID, GC-MS/0.3–0.4% (*v*/*w*) fresh material Resinoid obtained from the gum of labdanum using ethanol/2.9% of the dried material; 3.1% of the resinoid was the volatile fraction Concrete: extraction of dried material (17% moisture) with hexane and concentration under vacuum/5.0% from the dried material; 4.2% was the volatile fraction Absolute: obtained from the concrete using absolute ethanol with moderate heating followed by one night at −18 °C and the waxes eliminated by cold filtration/4.5% from the dried material; 4.6% was the volatile fraction	viridiflorol 19, bornyl acetate 17, camphene 12, ledol 8, α-pinene 5 labd-14-ene-16,18-diol 8, labd-14-ene-8,13-diol 5 pentyltricontane 18, labda-8,14-dienoic acid 10, labdanoic acid^#^ 9, labda-7,8-dienoic acid 7, labd-14-ene-16,18-diol 7, labda-8,20-dienoic acid 5, 16-kaurene 5, labda-8(20),13(16),14-triene 5, octadecane 5 labdenoic acid ^#^ 13, labdanoic acid ^#^ 9, labd-14-ene-8,13-diol 7, labd-14-ene-16,18-diol 6	Not determined	[29]
Morocco (Chefchaouen region (NW Morocco	Leaves	Hydro-distillation/GC-FID, GC-MS/1.4% (*v*/*w*)	1,8-cineole 19, viridiflorol 16, γ-terpinene 6	Not determined	[30]
Morocco (Tafoughalt, Eastern of Morocco)	Leaves	Hydro-distillation/GC-MS/0.14% (*w*/*w*) dried material	camphene 16, 2,2,6-trimethylcyclohexanone 7, borneol 11, terpinen-4-ol 6, δ-cadinene 6,	**Antioxidant activity** - Percentage of inhibition DPPH at 1–15 mg/mL: <60 and <80 (data from graphic)	[31]
Morocco (Oulmes)	Leaves	Hydro-distillation/GC/MS/0.1–0.2% (*v*/*w*) fresh material	camphene 18, verticiol 18, γ-gurjunene 7, *n*-butylcyclohexane ^§^ 6, bornyl acetate 6, 3-carene 5, *cis*-sabinol 5	**Antimicrobial activity** - MIC–MBC (mg/mL) *Escherichia coli* 25–25, *Pseudomonas aeruginosa* 25–25, *Staphylococcus epidermidis* 12.5–12.5, *Staphylococcus aureus* 6.25–6.25 **Antifungal activity** - MIC–MFC (mg/mL) *A niger* 0.001–0.001, *Trichophyton rubrum* 0.01–0.01, *C. albicans* 0.001–0.001	[32]
Morocco (Taza region)	Aerial parts	Hydro-distillation/GC-MS/not determined	viridiflorol 29, γ-gurjunene 15, cadina-1,4-diene 6, borneol 5	**Antimicrobial activity** - MIC (mg/mL) for *Clavibacter michiganensis subsp. michiganensis*: 0.78, *Pseudomonas savastanoi pv. savastanoi*: 0.57	[33]
Morocco (El Harcha forest, province of Khemisset, 150 km south-east of Rabat)	Unknown	Hydro-distillation/GC-FID/GC-MS/0.21% (*v*/*w*) dried material	viridiflorol 18, *trans*-pinocarveol 11, bornyl acetate 9, ledol 9, *p*-cymene 6, borneol 5, sclareol 5	**Antimicrobial activity** - MIC and MBC = 10 µL/mL for *S. aureus*, *Acinetobacter baumannii*, *E. coli*, *Salmonella typhi* **Antifungal activity** - MIC–MFC (µL/mL): *C. albicans*: 32–32; *C. tropicalis*: 64–64; *C. glabrata*: 32–32; *C. dubliniensis*: 32–32; *Candida* sp.: 16–62; *R. rubra*: 32–32; *C. neoformans*: 64–64; *Penicillium* sp.: 64–64; *Fusarium* sp.: 64–64; *A. niger*: 32–32	[34]
Morocco (Aknoul region)	Aerial parts	Microwave-assisted hydro-distillation/GC-MS/4.15% (*v*/*w*)	γ-terpinene 18, linderol * (C_10_H_18_O) 18, borneol 14, carvacrol 8, camphene 7, caryophyllene 7, β-cymene 5	**Antioxidant activity** - IC_50_, µg/mL: DPPH 178.29; ABTS 134.02; FRAP 321.71; β-carotene 246.14 **Antimicrobial activity** - MIC–MFC (%, *v*/*v*): *C. albicans* 1–2; *C. tropicalis* 8–16 - MIC–MBC (%, *v*/*v*); *Listeria innocua* 0.25–0.5; *E. coli* O157:H7 4–4; *Proteus mirabilis* 0.25–0.25 **Inhibition of α-amylase** - IC_50_ = 0.41 mg/mL **Inhibition of α-glucosidase** IC_50_ = 0.49 mg/mL **Inhibition of pancreatic lipase** IC_50_ = 0.004 mg/mL	[35]
Portugal (Douro region)	Leaves	Water followed by dichloromethane extraction/GC-MS-FID; NMR for 2,2,6-trimethylcyclohexanone/not determined	acetophenone 29, 2-phenylethanol 12, 2,2,6-trimethylcyclohexanone 12, borneol 8	Not determined	[36]
Portugal (wild in the mountains of the center-interior; cultivated in the north after propagation from wild plant found in the South of Portugal	Leaves and small branches	Hydro-distillation/GC-MS, GC-FID/0.2–0.3% dried material	Wild (fresh material): viridiflorol 15, α-pinene 5; dried material: viridiflorol 17, C_15_H_26_O sesquiterpene alcohol 6, globulol 5 Cultivated (fresh material): viridiflorol 15; dried material: viridiflorol 14, 15-nor-labdan-8-ol 5	Not determined	[37]
Portugal (Mirandela, Northern Portugal)	Leaves	Simultaneous distillation–extraction (SDE) (Likens-Nickerson): extraction solvent (pentane); extraction time (60 min)/headspace solid-phase microextraction (HS-SPME) for 60 min at 40 °C and with 20% NaCl using 85 µm polyacrylate fiber/GC-FID)/not determined	**mg/g (>3%):** α-pinene 22, 2,2,6-trimethylcyclohexanone 6, bornyl acetate 4, borneol 3	Not determined	[38]
Portugal (Bragança region, northeast Portugal)	Branches, in the floral stage	Hydro-distillation/GC-FID, GC-MS/not determined	2,2,6-trimethylcyclohexanone 30, viridiflorol 7	Not determined	[39]
Portugal (Universidade de Évora, Évora)	Leaves	Hydro-distillation/GC-FID, ^13^C-NMR/not determined	α-pinene 36, camphene 12, fenchone 9, bornyl acetate 9, viridiflorol 8	**Antimicrobial activity** - Inhibition zone [mm] *S. aureus* ATCC 25923 (11), *Bacillus subtilis* ATCC 6633 (11), *E. coli* ATCC 25922 (10), *P. aeruginosa* ATCC 27853 (9), *S. aureus* 42/60 (23), *E. coli* SA/4 (20) and *S. pneumoniae* ATCC 49619 (38)	[40]
Portugal (Mértola)	Obtained commercially from Herdade de Vale Covo; aerial parts particularly leaves	Steam distillation/GC-FID/GC-MS/not determined	α-pinene 39, camphene 8, *trans*-pinocarveol 6, bornyl acetate 5	**Antioxidant activity** - DPPH IC_50_ (%) = 0.9 - β-carotene bleaching IC_50_ (%) = 0.48 **Antimicrobial activity** - MIC (μL/mL) *S. aureus* ATCC 25923 (16), *L. monocytogenes* LMG 16779 (8), *E. faecalis* ATCC 29212 (8), *B. cereus* ATCC 11778 (2), *E. coli* ATCC 25922 (32), *S. Typhimurium* ATCC 13311 (32), *P. aeruginosa* ATCC 27853 (32)	[41]
Portugal (Beira Baixa)	Aerial parts of waste samples resulting from forest landscaping by Silvapor, Ambiente e Inovacao	Steam distillation using a stainless-steel distiller (1100 L, Vieirinox^®^, Aveiro, Portugal)/GC-FID, GC-MS/0.01% (March)–0.04% (*v*/*w*) (August)	March–August: α-pinene 28–25, camphene 25–13, limonene 7–1, tricyclene 5–2, bornyl acetate 4–6, viridiflorol 2–6	**Antimicrobial activity** - Diameter of the inhibition zone (mm): March–August *Escherichia coli* DSM 1077 9.3–12.0; *Staphylococcus aureus* ATCC 6538 8.5–9.8; *Candida albicans* 8.8–9.0 **Antioxidant activity** March–August - Percentage of inhibition at 30 µL (ABTS) 68.1–69.7 - Percentage of inhibition at 50 µL (Xanthine oxidase) 9.9–98.5	[42]
	Aerial parts of waste samples resulting from forest landscaping by Silvapor, Ambiente e Inovacao	Hydro-distillation/GC-FID, GC-MS/0.15% (*v*/*w*) (August)	August: α-pinene 13, camphene 5, *trans*-pinocarveol 5, viridiflorol 5	Not determined	
Portugal (central-west region of Portugal)	Flowers, leaves, and stems (commercially purchased from Aromas do Valado, Portugal)	Steam distillation/GC-MS/not determined	α-pinene 36, camphene 7, 2,2,6-trimethylcyclohexanone 7, *o*-cymene 5, bornyl acetate 5	**Ecotoxicity** Non-toxic because EC_50_ is >100 mg/L)	[43]
Portugal	Leaves, flowers, and thin branches, acquired from Proentia^®^ company (Portugal)	Steam distillation/GC-FID, GC-MS/not determined	α-pinene 50, camphene 10	**Antioxidant activity** - DPPH: IC_50_%, *v*/*v*) 0.691 **In vitro cytotoxicity** - Fibroblasts L929 IC_50_ (%, *v*/*v*) 0.027 Macrophages (RAW 264.7) IC_50_ (%, *v*/*v*) 0.012 NO production EC_50_ (%, *v*/*v*) 0.002 **Antimicrobial activity** - MIC-MLC (minimum lethal concentration) (%, *v*/*v*) *S. aureus* 1–2, *Staphylococcus epidermidis* 0.06–1, *E. coli* >2–>2, *P. aeruginosa* 2–2 *Cutibacterium acnes* 0.25–0.5 **Antifungal activity** - MIC–MLC (%, *v*/*v*) *C. albicans* 1–>2, *Aspergillus brasiliensis* 0.5–>2 ***C. acnes* biofilm formation**: Remarkable effect in inhibiting biofilm formation at all tested concentrations (1/4, ½, 1, 2 MIC) ***C. acnes* biofilm disruption**: Effect in inhibiting biofilm disruption at the concentrations (1/2, 1, 2 MIC)	[17]
Portugal	Leaves, flowers, and thin branches, acquired from Proentia^®^ company (Portugal)	Steam distillation/GC-FID, GC-MS/not determined	α-pinene 50, camphene 10	**Antimicrobial activity** - MIC–MBC (%, *v*/*v*) of *C. acnes* isolates of different phylotypes: 0.06–1.00 Half maximum effective concentration (EC_50_) for the effect on biofilm biomass—metabolic activity (%, *v*/*v*) of *C. acnes* isolates: 0.083–>0.938	[44]
Spain	Leaves and stalks	Distillation/GC-FID, GC-MS/not determined Enantio-GC-MS using the stationary phase 2,3-diethyl-6-tert butyl-β-cyclodextrin	**g/100 g**: α-pinene 24, camphene 13, α-copaene 8, bornyl acetate 6, *cis*-pinocamphone 6, limonene 4, *p*-cymene 4, α-terpineol 4, (*E*)-caryophyllene 3, *trans*-pinocamphone 3 Enantiomeric ratio: β-pinene 7 (−):93 (+), sabinene 23 (−):77 (+), camphor 52 (−):48 (+), linalool (92):8 (+), terpinen-4-ol 22 (−):78 (+), α-terpineol 85 (−):15 (+)	Not determined	[45]
Spain (Guadarrama mountain range (San Lorenzo del Escorial	Aerial parts	Hydro-distillation/GC-FID, GC-MS/0.34% fresh material	*trans*-pinocarveol 20, viridiflorol 14, bornyl acetate 7, terpinen-4-ol 6, 2(10)-pinen-3-one 5, *p*-mentha-1,5-dien-8-ol 5, α-pinene 5	**Phytotoxicity** - Weed seed germination percentages at 0.125–1 µL/mL: *Amaranthus hybridus* 0–0%, *Portulaca oleracea* 90.0–41.0%, *Chenopodium album* 23.0–33.0%, *Conyza canadensis* 1.0–1.0%, *Parietaria judaica* 1.0–1.0% - Seedling length (mm) at 0.125–1 µL/mL: *A. hybridus* (=no seedling length); *C. canadensis* 0.25–0.07; *P. judaica* 0.61–0.39	[46]
Spain (mountainous area near Puertollano, Ciudad Real)	Aerial parts	HS-SPME using a 50/30 µm divinylbenzene (DVB)/polydimethylsiloxane (PDMS) fiber/GC-MS/not determined	α-pinene 17, bornyl acetate 15, camphene 13, 2,2,6-trimethylcyclohexanone 12, α-campholenal 5, *trans*-pinocarveol 5	Not determined	[47]
Spain (commercial provided by FLORAR s.r.l. (Pisa, Italy)	Aerial parts	GC-MS/not determined	α-pinene 45, *p*-cymene 6, unknown 6, *trans*-verbenol 5, bornyl acetate 5	**Cytotoxicity** - IC_50_ (ppm) for the different cell lines: human chronic myelogenous erythroleukemia (K562 = 46.9), human breast adenocarcinomas (MCF7 = 90, MDA-MB-231, T47D > 300), human neuroblastoma cell lines derived from a highly malignant tumor cells (SH-SY5Y = 92.8), endometrial stromal cells (ESCs = 264.7)	[48]
Spain (Andévalo in Huelva and Cerezal in Zamora)	Branches with a maximum stem diameter of 50 mm that included twigs, leaves, and fruits	Steam distillation/GC-MS/<0.1% (*w*/*w*) dried material	Andévalo–Cerezal: α-pinene 43–19, viridiflorol 13–24, ledol 4–7, bornyl acetate 4–5, camphene 2–7	**Antimicrobial activity** - MIC–MBC (mg/mL) (Andévalo) *Escherichia coli* 0.6–0.6, *Klebsiella pneumoniae* >2.5–> 2.5, *Morganella morganii* 2.5–2.5, *Proteus mirabilis* >2.5–>2.5, *Pseudomonas aeruginosa* 2.5–> 2.5, *Enterococcus faecalis* 1.25–1.25, *Listeria monocytogenes* 0.6–0.6 - MIC-MBC (mg/mL) (Cerezal) *E. coli* 0.6-0.6, *K. pneumoniae* >2.5–> 2.5, *M. morganii* 0.6–0.6, *P. mirabilis* >2.5–>2.5, *P. aeruginosa* 2.5–> 2.5, *Enterococcus faecalis* 0.6–0.6, *L. monocytogenes* 0.3–0.3 **Antioxidant activity** - Reducing power assay (effective concentration at which the absorbance is 0.5 and achieving 50% of antioxidant potential) (EC_50_) (mg/mL) Andévao–Cerezal 1.64–1.30 **Antioxidant activity in cell cultures** - Cellular murine macrophage cell line (RAW 264.7) antioxidant activity in % oxidation inhibition at the maximum concentration of 2 mg/mL: Andévao–Cerezal: 83.24–81.13 **Cytotoxicity** - activity in GI_50_ (concentration of the extract causing 50% of cell growth inhibition (µg/mL): Andévalo–Cerezal NCI-H460 (lung carcinoma): 14.27–53.80, MCF-7 (breast carcinoma): 27.80–58.45, AGS (gastric carcinoma): 78.41–46.59, CaCo2 (colon carcinoma): 75.31–48.78, Two normal cell lines: PLP2 (porcine liver cells): 207.64–142.08, VERO (monkey kidney cells): 70.77–46.03 using the sulforhodamine B (SRB) assay - **Anti-inflammatory activity** Inhibition of the lipopolysaccharide (LPS)-induced NO (nitric oxide) production on RAW264.7 (IC_50_) (µg/mL): Andévalo–Cerezal 19.27–21.00	[49]
Spain (Guadalajara, 1030 m above sea level))	Aerial parts in bales at different storage times (1–120 days)	Steam distillation/GC-FID, GC-MS/0.075% (0–7 days of storage); 0.066% (15–30 days of storage); 0.052% (*w*/*w*) (100–120 days of storage)	- 0–7 days of storage: α-pinene 50, viridiflorol 10 - 15–30 days of storage: α-pinene 47, viridiflorol 12 - 100–120 days of storage: α-pinene 47, viridiflorol 13	Not determined	[50]
Spain (central or northern locations)	Twigs	Steam distillation/GC-FID, GC-MS/0.036% (September 2018); 0.037% (*w*/*w*) (October 2019)	- September 2018–October 2019: α-pinene 52–39, viridiflorol 6–10	**Antioxidant activity** - Oxygen radical absorbance capacity (ORAC) assay expressed as µmoL Trolox/g EO: 209.36 (October 2019); <200 (September 2018)	[51]
Spain (Extremadura)	Aerial parts	Hydro-distillation/GC-MS/0.349% (v/w) dried material	ledol 19, α-pinene 15, viridiflorol 7, bornyl acetate 5	**Antifungal activity** - Effective dose 50 in logit analysis (ED50) µL/mL): *Cryphhonectria parasitica* 0.027, *Fusarium oxysporum* 0.033, *Phytophthora cinnamomi* 0.024, *Rhizoctonia solani* 0.017 **Phytotoxicity** - on *Raphanus sativus* (Germination index, GI = 1 µL dose) and *Lupinus luteus* seeds (GI = 4 µL dose)	[52]
Spain (Extremadura, different places)	Aerial parts	Hydro-distillation/GC-MS/0.19-0.42% (v/w) dried material	Pozuelo de Zarzón (slate) A1, A2, A3 viridiflorol 21, 18, 23; ledol 9, 9, 10; α-pinene 4, 6, 3; *trans*-pinocarveol 4, 5, 3 Valverde del Fresno (slate) B1, B23, B3 viridiflorol 21, 22, 21; ledol 11, 10, 10 Torre de Don Miguel (granite) C1, C2, C3 viridiflorol 18, 19, 20; ledol 10, 9, 9; α-pinene 6, 5, 6; *trans*-pinocarveol 5, 5, 5 Villasbuenas de Gata (granite) D1, D2, D3 viridiflorol 19, 20, 19; ledol 10, 8, 8; α-pinene 5, 7, 6; (*E*)-pinocarveol 4, 5, 5	**Phytotoxicity** Effect on seed germination and seedling length of *Raphanus sativus* L. - Germination inhibition ED_50_ (µL/mL) A1 0.025, A2 0.044, A3 0.033, B1 0.033, B2 0.034, B3 0.028, C1 0.055, C2 0.059, C3 0.049, D1 0.040, D2 0.048, D3 0.051 - Seedling length inhibition ED_50_ (µL/mL): 0.017–0.053	[53]
Spain		Hydro-distillation/GC-FID/GC-MS/0.03–0.19% (7-year-old populations; 0.001–0.12% (*w*/*w*) (12-year-old populations) dried material	7-year-old populations 2021 26/07, 17/08, 30/08, 13/09, 27/09, 18/10, 02/11, 15/11, 29/11, 13/12, 27/12 2022 17/01, 03/02, 28/02, 28/03, 24/04, 30/05, 27/06 α-pinene 43, 35, 45, 52, 58, 58, 61, 58, 60, 58, 50, 53, 50, 47, 50, 49, 39, 33 viridiflorol 4, 10, 6, 5, 4, 3, 3, 3, 3, 3, 4, 4, 4, 4, 4, 4, 4, 7 *allo*-aromadendrene + *trans*-pinocarveol 5, 6, 6, 4, 4, 4, 4, 4, 3, 4, 4, 4, 4, 5, 5, 4, 4, 4 bornyl acetate 4, 4, 3, 3, 3, 3, 2, 2, 2, 3, 3, 3, 3, 4, 2, 2, 4, 5 12-year-old populations 2021 26/07, 17/08, 30/08, 13/09, 27/09, 18/10, 02/11, 15/11, 29/11, 13/12, 27/12 2022 17/01, 03/02, 28/02, 28/03, 24/04, 30/05, 27/06 α-pinene 43, 16, 19, 55, 61, 52, 60, 58, 58, 59, 58, 56, 52, 44, 46, 44, 35, 27 viridiflorol 6, 17, 17, 5, 4, 4, 3, 3, 3, 4, 3, 4, 3, 5, 5, 6, 6, 13 *allo*-aromadendrene + *trans*-pinocarveol 5, 6, 6, 4, 3, 4, 3, 3, 3, 3, 3, 3, 3, 4, 4, 4, 4, 4 bornyl acetate 3, 6, 8, 3, 2, 3, 3, 3, 3, 3, 2, 2, 3, 3, 3, 3, 4, 5	**Antioxidant activity** - ORAC method, IC_50_ µg/mL 117.47–>256.00 **Antimicrobial activity** against *Bacillus cereus*: - MIC Broth–MIC agar (µg/mL) 512–>1024	[54]
Spain (Extremadura)	Aerial parts (stems, leaves, flowers, and fruits)	Hydro-distillation/GC-MS/0.17% (*v*/*w*) dried material (plants in flowering phase); 0.27% (*v*/*w*) dried material (plants in fruit maturation)	Plants in flowering phase of 3–5 years; 9–11 years; >15 years viridiflorol 20, 20, 17; ledol 10, 8, 8; 2,2,6-trimethylcyclohexanone 4, 5,5 Plants in fruit maturation of 3–5 years; 9–11 years; >15 years viridiflorol 20, 20, 20; ledol 10, 9, 10; α-pinene 5, 5, 4	**Phytotoxicity** - Seedling length inhibition (%) of *R. sativus* for EO doses 0.011, 0.022, 0.044, 0.088, 0.178 µL/mL: 16.4–98.0 - Seed germination inhibition (%) of *R. sativus* for EO doses 0.011, 0.022, 0.044, 0.088, 0.178 µL/mL: 6.3–89.1 - Seedling length inhibition (%) of *R. sativus* for EO doses 0.011, 0.022, 0.044, 0.088, 0.178 µL/mL: 35.8–99.2 - Seed germination inhibition (%) of *R. sativus* for EO doses 0.011, 0.022, 0.044, 0.088, 0.178 µL/mL: 4.0–94.9	[55]
Spain (Extremadura)	Aerial parts (stems, leaves, flowers, and fruits)	Hydro-distillation/GC-MS/not determined	August–October: Viridiflorol 20–16, α-pinene 3–13, ledol 9–7	Germination index (GI) of seeds of rice and tomato (0%): 0.08 µL/mL GI of seeds of *Echinochloa crus-galli* (0%): 0.08 µL/mL	[56]
Spain (Ciudad Real)	Mature leaves nascent in the spring and exuding high levels of labdanum during the summer	Supercritical carbon dioxide (8–10 MPa and 30–60 °C/GC-FID/between 40 and 60 °C at a fixed pressure of 8 MPa; extraction yields decreased as temperature increased, but there was no significant change between 30 and 40 °C. Oil extraction yields rise as pressure increases at a constant temperature of 40 °C. Similar pattern at 30, 50, and 60 °C. The best conditions for obtaining the profile reported were temperature (T) = 40 °C; pressure (P) = 9 MPa; flow rate = 0.7 hg/h; diameter of particle = 0.30 mm	Extraction times (15, 30, 60, 90, 180 min): Terpenes: camphor 25, 23, 22, 22, 22; α-pinene 20, 20, 20, 20, 19; camphene 5, 4, 4, 4, 4; borneol 5, 5, 5, 4, 4; γ-terpineol 5, 5, 5, 5, 5; thymol 5, 4, 4, 4, 4. Waxes: nonacosane 21, heptacosane 14, tricosane 12, pentacosane 9, methyleicosane 7, entriacontane 7, triacontane 6, hexacosane 5	Not determined	[57]
Unknown	Commercially provided by Lemondarome Co., Std. Aerial parts	Steam distillation/GC-MS-MS/not determined	camphene 32, α-pinene 16, bornyl acetate 16, tricyclene 7, 2,2,6-trimethylcyclohexanone 7	**Antioxidant activity** - mg Trolox equivalent (TE)/g: DPPH, ABTS, Cuprac, FRAP: 6.82, 12.10, 57.20, 38.30 Antioxidant activity phosphomolybdenum (mmolTE)/g): 13.42 Antioxidant activity chelating activity (mg EDTA/g): 24.31 **Antimicrobial activity** - MIC (mg/mL). *Proteus mirabilis* 50, *B. subtilis* >50, *S. aureus* 50, *C. albicans* 12.5 **Enzyme inhibitory activity** - α-glucosidase (µg acarbose equivalent/g) 1.39; -α-amylase (µg acarbose equivalent/g) 1.07; tyrosinase (mg kojic acid equivalent/g) 10.22; butirylcolinesterase (mg galantamine equivalent/g) 2.45; acetylcholinesterase (mg galantamine equivalent/g) 4.71	[58]

^13^C-NMR; carbon 13 nuclear magnetic resonance; ABTS: 2,2′-azino-bis(3-ethylbenzothiazoline-6-sulfonic acid; DPPH: 2,2-diphenyl-1-picrylhydrazyl; FRAP: Ferric-reducing antioxidant power; GC-FID: gas chromatography–*flame ionization detection*, GC-MS: gas chromatography–mass spectrometry; IC_50_: concentration of drug required for 50% inhibition; MIC: minimum inhibitory concentration; MBC: minimum bactericidal concentration; ORAC: oxygen radical absorbance capacity. * It is a compound generally not detected in EOs. ^#^ These compounds are unknown. Although they may be regarded as labdanolic acid, the authors also stated that labdanolic acid was detected in their samples, however, in considerably smaller amounts. ^§^ Possible artifact. ● Whenever the concentration is not expressed as a percentage, it is indicated in bold in the appropriate place.

**Table 2:** Chemical composition and biological properties of *C. ladanifer* hydrolates.

Origin	Plant Part Used	Extraction Type/Identification	Main Compounds (>5%)	Biological Properties	Reference
Portugal (Beira Baixa	Aerial parts of waste samples resulting from forest landscaping by Silvapor, Ambiente e Inovacao	Steam distillation using a stainless-steel distiller (1100 L, Vieirinox^®^, Aveiro, Portugal)/GC-FID, GC-MS Hydro-distillation/GC-FID, GC-MS	March–August: *trans*-pinocarveol 5–13, 2,6,6-trimethyl cyclohexanone 12–9, borneol 9–7, terpinen-4-ol 6–2, 1-methyl cycloheptanol ^§^ 3–5, verbenone 5–1, August: *trans*-Pinocarveol 8, verbenone 8, 1-methyl cycloheptanol ^§^ 5	**Antimicrobial activity** -Diameter of the inhibition zone (mm): March–August; *E. coli* DSM: no activity; *S. aureus* ATCC 6538: no activity **Antioxidant activity** March–August: Percentage of inhibition at 30 µL (ABTS) 8.2–8.1; percentage of inhibition at 60 µL (superoxide) 14.3–13.4; percentage of inhibition at 50 µL (xanthine oxidase): 25.3–25.7; percentage of inhibition at 200 µL (chelating activity): 24.1–25.1 **Anti-inflammatory activity** Albumin denaturation assay: Percentage of inhibition at 1 mL hydrolate: 94% for both samples Not determined	[42]
Portugal (central-west region of Portugal)	Flowers, leaves and stems (commercial purchased to Aromas do Valado, Portugal)	Steam distillation/GC-MS	4-hydroxy-3-methylacetophenone 22, *p*-cymen-8-ol 11, myrtenol 11, D-verbenone 10, endo-borneol 8	**Ecotoxicity**: No observable effect on *Daphnia magna* after 48 h of exposure up to 2000 mg/L.	[43]
Spain (Extremadura)	Aerial parts	Hydro-distillation/GC-MS	*trans*-pinocarveol 11, 2,2,6-trimethylcyclohexanone 10, pinocarvone 6, ledol 5	**Antifungal activity** Effective dose 50 in logit analysis (ED_50_) µL/mL): *C. parasitica* 141.9; *F. oxysporum* 235.2; *P. cinnamomi* 144.4; *R. solani* 88.1 **Seedlings mortality** (%) of the *Lupinus luteus* caused by three strains of *P. cinnamomi* (CA-4, CA-9, MYC-18) at the end of phase at 2 at 0, 30, 62.5, 125, 250, 500 µL/mL of hydrolate: (0): 100, 100, 100; (30): 100, 100, 90; (62.5): 100, 100, 90; (125): 90, 100, 100; (250): 50, 30, 10; (500): 0, 0, 0 ED_50_ (µL/mL): 234.1, 238.1, 165.5	[52]
Portugal	Leaves, flowers, and thin branches acquired from Proentia^®^ company (Portugal)	Steam distillation/GC-FID, GC-MS	*trans*-pinocarveol 25, borneol 14, terpinen-4-ol 10, 2,6-trimethylcyclohexanol * 8, 1,8-cineole 6, myrtenol 6, acetophenone 5, verbenone 5	**Antioxidant activity** DPPH: IC_50_%, *v*/*v*) 43.00 (poor due to the low antioxidant activity index 0.0037) **In vitro cytotoxicity (%, *v*/*v*)** Fibroblasts L929: 5.71; Macrophages: 8.57; NO production (EC50): 0.79 **Antimicrobial activity** MIC-MLC (minimum lethal concentration) (%, *v*/*v*): *S. aureus* *Staphylococcus epidermidis*, *E. coli* *P. aeruginosa*: Does not present relevant antimicrobial activity *Cutibacterium acnes*: 25	[17]

ABTS: 2,2′-azino-bis(3-ethylbenzothiazoline-6-sulfonic acid; DPPH: 2,2-diphenyl-1-picrylhydrazyl; GC-FID: gas chromatography–flame ionization detection*,* GC-MS: gas chromatography–mass spectrometry; IC_50_: concentration of drug required for 50% inhibition; MIC: minimum inhibitory concentration; MBC: minimum bactericidal concentration. * It appears that there was an error. ^§^ Possible artifact.

## Data Availability

No new data were created or analyzed in this study.
